# New trends in detection of harmful insects and pests in modern agriculture using artificial neural networks. a review

**DOI:** 10.3389/fpls.2023.1268167

**Published:** 2023-11-02

**Authors:** Dan Popescu, Alexandru Dinca, Loretta Ichim, Nicoleta Angelescu

**Affiliations:** ^1^ Faculty of Automatic Control and Computers, University Politehnica of Bucharest, Bucharest, Romania; ^2^ Faculty of Electrical Engineering, Electronics, and Information Technology, University Valahia of Targoviste, Targoviste, Romania

**Keywords:** insect detection, pest detection, precision agriculture, image processing, deep learning, artificial neural networks

## Abstract

Modern and precision agriculture is constantly evolving, and the use of technology has become a critical factor in improving crop yields and protecting plants from harmful insects and pests. The use of neural networks is emerging as a new trend in modern agriculture that enables machines to learn and recognize patterns in data. In recent years, researchers and industry experts have been exploring the use of neural networks for detecting harmful insects and pests in crops, allowing farmers to act and mitigate damage. This paper provides an overview of new trends in modern agriculture for harmful insect and pest detection using neural networks. Using a systematic review, the benefits and challenges of this technology are highlighted, as well as various techniques being taken by researchers to improve its effectiveness. Specifically, the review focuses on the use of an ensemble of neural networks, pest databases, modern software, and innovative modified architectures for pest detection. The review is based on the analysis of multiple research papers published between 2015 and 2022, with the analysis of the new trends conducted between 2020 and 2022. The study concludes by emphasizing the significance of ongoing research and development of neural network-based pest detection systems to maintain sustainable and efficient agricultural production.

## 1 Introduction

The adoption of artificial intelligence (AI) and integrated structures has rapidly become multidisciplinary and spread across various fields, dominating research areas and plans in previous years (Zhang, 2022). Thanks to the technological advancements in the field of AI and more importantly in the field of deep learning (DL), a multitude of domains enjoy notable results for various associated tasks (Abade et al., 2021). This advance has brought such technologies to the fore with great success, its upward trajectory and continued development being supported by a range of technological, financial, and educational resources (Kumar and Kukreja, 2022). The integration of AI and integrated structures has significantly impacted insect pest detection, offering innovative solutions to this pressing agricultural and environmental concern. This evolution is driven by advancements in DL and supported by substantial resources, ultimately resulting in the development of highly efficient and sustainable techniques for insect pest detection and management (LeCun et al., 2015).

Considering the agricultural field, these techniques have enjoyed great popularity and started to be adopted on a large scale, where human labor does not have the necessary time and speed to analyze the data in a timely manner and to cover considerable areas in the monitoring area (De Cesaro Júnior & Rieder, 2020). Often, these features are more than useful and relevant to every operation, and early detection, monitoring, and classification deliver results to match (Ampatzidis et al., 2020). Due to this aspect, automation areas have been successfully introduced and are based on thorough research and massive development and optimization techniques (Ahmad et al., 2022). Technological advances, particularly in deep learning (DL), have been critical in the identification of insect pests. These breakthroughs have resulted in tremendous progress in correctly detecting and managing insect pests in agriculture and other industries. In recent years, the use of artificial intelligence (AI) and integrated structures has spread to a variety of disciplines, with a special emphasis on insect pest identification. This integrative approach has gained prominence in research agendas, altering how we address pest-related concerns.

Intelligent and precision techniques are necessary for farmers, especially for automation, because they reduce the complexity of pest detection and counting estimation, compared to a process done manually by farmers or authorized auxiliary persons, this process being expensive and requiring a lot of time execution (Ahmad et al., 2018; Apolo-Apolo et al., 2020; Thakare and Sankar, 2022). Solutions based on DL and the automation of the processes involved in crop management prove to be effective, with high coverage and low costs (Iost Filho et al., 2019). At the same time, it helps the process of detecting and managing pests in a timely manner, without resorting to highly invasive solutions and representing effective measures (Mavridou et al., 2019).

Considering the chemical treatment applied with pesticides, the amounts administered become directly proportional to the degree of infestation and do not present sustainability characteristics, as they are present or required in modern development areas. Pest populations cause massive, considerable damage to crops of various types and sizes. This highlights an important point because agriculture is the most significant economic branch in many countries (Cardim Ferreira Lima et al., 2020). Monitoring, managing, and protecting crops from insect pests is an important step and an area of thorough research (Zhu et al., 2020). In an unfortunate setting, the productivity and production volume of agricultural areas is strongly affected by the appearance and presence of pests and their widespread (Ahmad et al., 2022). The identification and monitoring of pests, mostly represented by insects, and careful management of crops are of interest in agricultural development. Many times, the management of these pests takes place in poorly managed processes, without clear expertise, and often based on invasive, non-sustainable, and polluting solutions (Wen & Guyer, 2012). Modern models and techniques based on AI and DL, especially image processing and convolutional neural networks (CNNs), are very useful and effective in the so-called precision agriculture (PA) or integrated pest management (IPM) (Mavridou et al., 2019). The way to combine automatic or supervised image acquisition using drones and digital cameras with the emphasized developments of models based on CNNs was a great success (Du et al., 2022; Zhang et al., 2022).

The continuous progress of DL models has brought to the fore several notable applications for pest management and PA in general. CNNs, as part of DL, represent a state-of-the-art around image analysis and are mainly and successfully used for the development of classification, object detection, or segmentation tasks (Wang et al., 2017; Zhang et al., 2020). In principle, the convolution techniques and the mathematical models present among them make possible the existence and continuous expansion of the previously mentioned techniques and even their strong development, modification, or optimization. Starting from an initial and innovative step, these types of techniques have been developed and researched along the way, having today a series of remarkable architectures with adequate performance in various tasks (Tian H. et al., 2020; Zhang et al., 2022). The study (Nanni et al., 2022) addresses the problem of automatic identification of invasive insects to combat crop damage and losses. The authors created ensembles CNNs using various topologies optimized with different Adam variants for pest identification. The best ensemble, combining CNNs with various Adam variants, achieved impressive results, surpassing human expert classifications on several known datasets. With the awareness that agricultural pests severely impair food crop quality, the importance of agriculture as an economic backbone is underlined in (Sanghavi et al., 2022). Machine learning models have been employed to handle pest categorization and detection, however they suffer when dealing with insects that have similar traits but live in diverse environments. The paper offers an enhanced deep learning model named Hunger Games Search-based Deep Convolutional Neural Network (HGS-DCNN) for efficient insect identification with improved accuracy to address this difficulty. The process of recognizing and classifying insects, addressing several challenges, was proposed by the authors in paper (Xia et al., 2018) locating information on an insect quickly as part of a complex backdrop, precisely recognizing insect species, especially when they are highly similar within the same species (intra-class) and across species (inter-class) and identifying differences in the appearance of the same insect species at various stages of development. These issues are crucial in the field of insect recognition and categorization.

Starting with a motivation area, we highlighted IPM and PA for this study. There are several problems facing the current agricultural sector in terms of production management, security, and the negative impact of external and biological/natural factors (Csillik et al., 2018; Ronchetti et al., 2020). Speaking of the agricultural area, the desire for sustainability has brought to the fore a series of characteristics represented by IPM and a series of actions for the areas where it can be applied. Basically, IPM represents a collection of good practices to attract attention and give rise to effective approaches in the fight against pest populations and for the optimal and timely management of the associated effects (Cardim Ferreira Lima et al., 2020). IPM has developed over the years based on up-to-date, well-verified information and gradual adoption. A series of studies developed and researched this topic in detail for the construction of PA areas, with innovative and well-documented techniques (Velusamy et al., 2022). Moreover, the desire for sustainability quickly accentuated this. The accuracy of the information, the continuous monitoring, and the effective IPM documentation make possible the emergence and continuous support of good practices that can be successfully applied to the development of the agricultural field (Ronchetti et al., 2020; Misango et al., 2022). In principle, the adoption of IPM is done for the adequate control of pests and to reduce them and their effects to a tolerable level. On the other hand, the IPM effect also has a considerable positive impact on the environment and the population. The desire for adoption is primarily emphasized by the decrease in the amounts of pesticides used after prior monitoring. The effects of pests, their presence, and plant diseases represent a serious threat to agricultural production and the resulting food security due to the agricultural sector (Misango et al., 2022; Wu et al., 2019). The IPM objective is to create a combination of actions associated with good practices to develop specific solutions for each agricultural area and culture. Although IPM notions and application methods are not relatively new techniques, a considerable number of studies have emerged to identify the status and trends of the agricultural sector regarding the existence of these good practices that IPM wishes to highlight.

As highlighted by the authors (Damos, 2015) the management of pests in a sustainable or ecological way brings into question the reduction of pesticides and the adoption of alternatives for the control and development of production in a safe and ecological way. Being a basic field, agriculture represents a sector that has enjoyed a series of changes over time marked by automation, modern crop management and monitoring models, and various smart methodologies. Research developed by the authors (Deguine et al., 2021) shows the impact and evolution of IPM practices over the last five or six decades. Data needed for the area of crop profiles, pesticides, and strategy plans for the safe management of agricultural areas were noted by (Bouroubi et al., 2022) to highlight an educational basis for decision-making and risk assessment. data creation and documentation were noted as necessary and examples of databases and applications that can be used for continuous and quality information with high availability were highlighted. The need for access to data and the influence of IPM adoptions were also noted by the authors (Tong et al., 2022) for the agricultural production area. Here, several mechanisms and factors for the adoption of good practices by farmers and the attached IPM notions, as well as research trends in these directions, have been noted. In a more advanced framework, the authors of the meta-analysis (Sekabira et al., 2022) emphasized socio-economic factors with impact in the combined area of IPM and climate-smart CS-IPM. To ensure the sustainability of agricultural ecosystems, the authors analyzed and noted the strategic determinants for the adoption of smart innovations in the case of modern agriculture and environmental policies. CS-IPM involves a range of practices and techniques that are tailored to local conditions and needs. These include crop diversification, conservation agriculture, integrated pest management, and the use of climate-resilient crop varieties.

Modern agriculture has great potential and is aided today by several powerful working and monitoring technologies to increase productivity, efficiency, and the eco-friendliness that can be attached. Precision farming techniques and advanced methodologies have helped to increase food security and environmental sustainability (Wen and Guyer, 2012). Analyzing the papers highlighted for this study, there is a general trend of massive adoption of technological processes or automation in the agricultural area as part of the idea and methods involved in PA. It uses data and precision farming tools such as sensors, drones, and precision planting equipment to gather information about soil, weather, and crop growth, and then use that information to make precise, data-driven decisions about planting, fertilizing, harvesting crops or pest detection and management (Popescu et al., 2020). This can help farmers to increase yields, reduce costs, and improve the efficiency of their operations, being a major advantage to achieve modern targets such as sustainability and ecological production.

**Figure 1:**
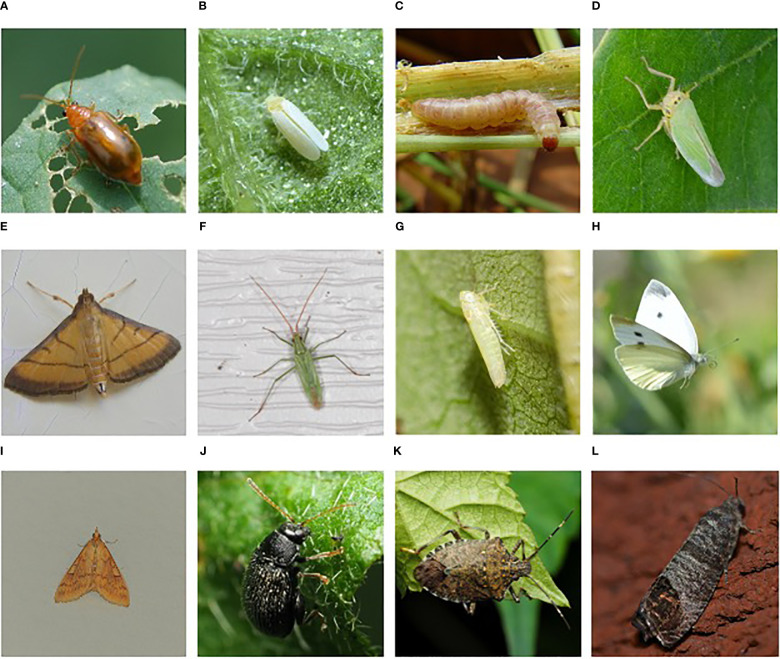
Examples of harmful insects for agriculture: **(A)** Aulacophora indica, **(B)** Bemisia tabaci, **(C)** Sesamia inferens, **(D)** Cicadella viridis, **(E)** Cnaphalocrocis medinalis, **(F)** Trigonotylus caelestialium, **(G)** Emposca flavenscens, **(H)** Pieris rapae, **(I)** Ostrinia nubilalis, **(J)** Epitrix fuscula, **(K)** Halyomorpha halys, **(L)** Cydia pomonella (Xie et al., 2018), (https://www.dlearningapp.com/web/DLFautoinsects.htm).

CNN-based systems for insects and pest detection have been successfully applied to a range of crops, including vegetables, fruits, and grains. In addition to identifying insects, CNNs can also detect damage caused by insects, such as holes and discoloration on plant leaves. This information can be used to quantify the severity of insect infestations and to guide pest management strategies. Digital images of plants and crops are obtained using cameras or drones equipped with high-resolution sensors. These images are then analyzed using CNNs, that has been shown to be highly effective at image classification and object detection tasks. However, the models used for monitoring need training and validation of insect pest datasets and innovative optimizations. Examples of digital images for this topic, illustrating several known insect pests, are shown in **Figure 1**: A) Aulacophora indica, B) Bemisia tabaci, C) Sesamia inferens, D) Cicadella viridis, E) Cnaphalocrocis medinalis, F) Trigonotylus caelestialium, G) Emposca flavenscens, H) Pieris rapae, I) Ostrinia nubilalis, J) Epitrix fuscula, K) Halyomorpha halys, and L) Cydia pomonella.There are often problems in the highly accurate detection of insects of interest, as they are part of the natural setting where the conditions in which these insects are captured are not optimal – accurate detection is hindered by lighting conditions, various artifacts, or obturations of various types (leaves, flowers, branches, fruits). Based on these limitations, there has been continuous research and development aimed at creating innovative techniques for extracting information of interest from digital images that illustrate real contexts.

The presence of natural factors with a negative impact on performance inclined toward the development of research based on concrete work methods. The general workflow for insect detection and monitoring in modern agriculture using neural networks is composed of the following phases: a) Data collection, b) Data processing, c) NN training, and d) Validation and testing.

To start developing a system for insect pest detection using digital images and CNNs, the first step is to collect relevant data consisting of images of insects and crops, which would need to be labeled and categorized to identify the type of insects encountered (Partel et al., 2019; Nanni et al., 2022). The next step is data preprocessing, which involves removing noise, distortion, or other anomalies from the collected data (Du et al., 2022). This can include resizing images, adjusting brightness and contrast, and data augmentation (Ahmad et al., 2022). A great feature extraction model can make use of DL techniques to focus attention on insect pests (Li et al., 2020; Li et al., 2022). From the preprocessed dataset, a representative subset of images needs to be selected for training CNNs to identify and classify insect pests in the images and adjust internal weights to improve accuracy (Khanramaki et al., 2021). Once trained, the CNN must be validated and tested on a separate dataset to evaluate its accuracy and identify any issues that need to be addressed. Since manual classification and detection are time-consuming automation using CNNs is preferred (Butera et al., 2021).

This paper wants to present a detailed review of the methods of automatic identification of populations of harmful insects by involving algorithms in the field of neural networks (NNs). Recently, it has been observed that the use of digital tools and services for the early and automatic detection of populations of harmful insects represents an impact factor on agricultural areas. Moreover, the optimization of agricultural processes in combination with these tools offers optimal and high-performance solutions. To facilitate reading the article, a list of abbreviations is given in Annex 1.

The presentation of the selected studies brings to the fore a series of key, modern methods related to the topic attached to the paper. Pest detection methods have made significant advancements over the years, but there are still several challenges and areas that need improvement in existing approaches. These challenges often include accuracy and reliability, data quality and quantity, integration with pest management, automation and scalability, real-time detection and species and diversity. Many current methods for pest detection still suffer from high rates of false positives (identifying non-pests as pests) or false negatives (failing to detect pests when they are present). On the other hand, developing accurate machine learning models for pest detection often requires large amounts of high-quality labeled data, which can be expensive and time-consuming to obtain. Imbalanced datasets, where certain pests are rare or hard to find, can lead to biased models that perform poorly on underrepresented pests. Pest species can be highly diverse, and methods that work for one pest may not be effective for others. Developing generalized detection methods that can adapt to different pests is a challenge.

## 2 Materials and methods

### 2.1 Investigation of references

The paper considered method workflow from PRISMA guidelines (Page et al., 2021) for insect detection and monitoring in agriculture based on NNs by investigating articles published between 2015 and 2022. This review article aims to provide an overview of the new trends and advancements in CNN research for insect pest detection in agriculture between 2015 and 2022. To select the papers for this review, the focus was primarily on papers that contribute to the development of CNN-based systems for insect pest detection in agriculture. Specifically, papers that propose novel CNN architectures, explore the use of transfer learning for insect pest detection, or apply CNNs to new insect pest detection tasks were prioritized. The selected papers demonstrate the power of CNNs in various applications for insect monitoring in modern agriculture, including object detection, segmentation, and recognition.

**Figure 2:**
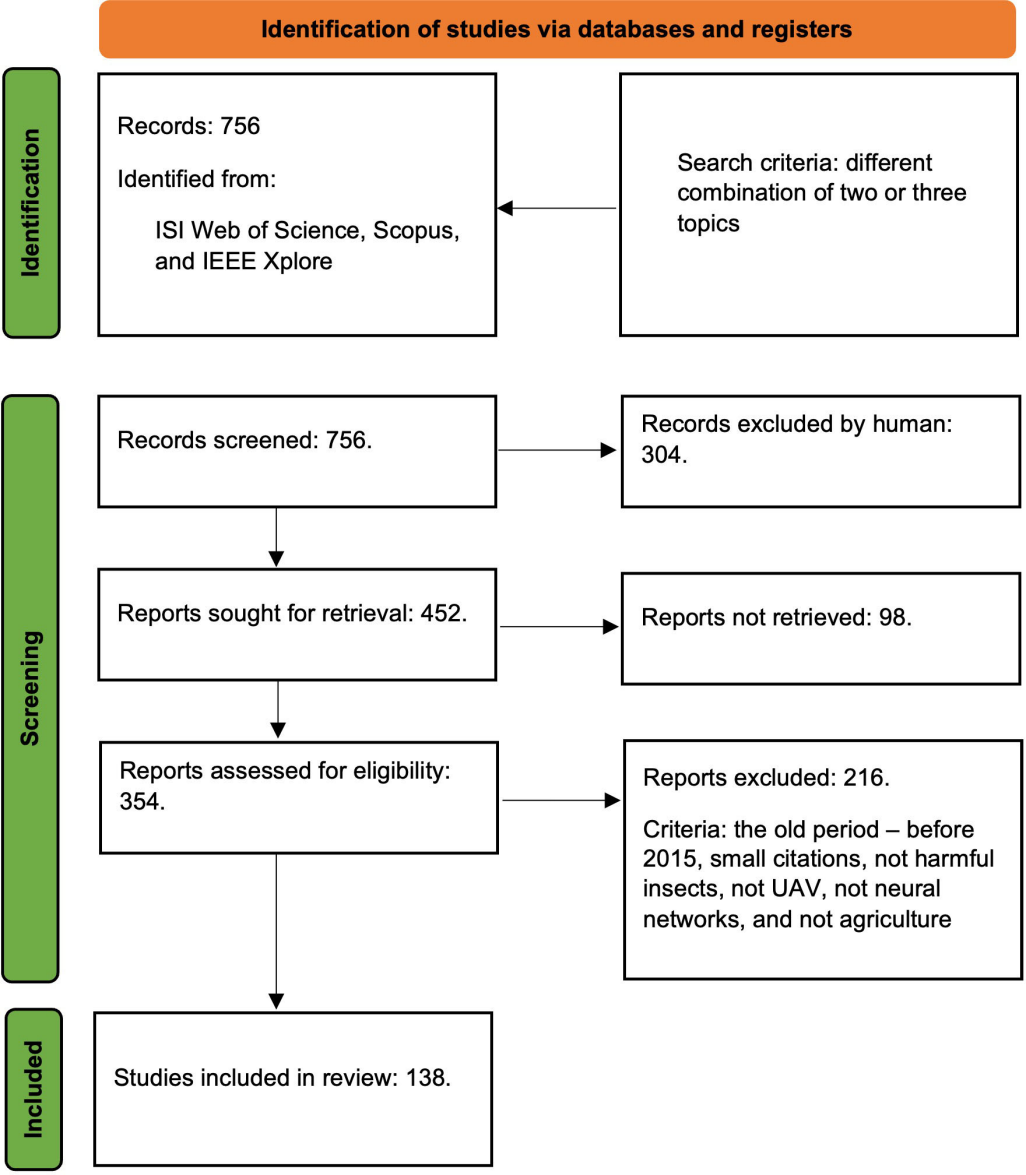
PRISMA 2020 flow diagram for this study.

The research databases used in this review were: Web of Science, Scopus, and IEEE. Following the Prisma flow diagram (**Figure 2**), several criteria were attached for searching and extracting articles of interest. Although there was an initially large number of papers identified for the topic of this review, the initial selection criteria extracted approximately 354 relevant studies in the first instance. Of all these, only 138 were chosen based on the final criteria related to new periods, new trends, attachment in top publications, and innovation. An initially large number of diverse research for the modern agricultural area and a considerable evolution in recent years are observed.

**Figure 3:**
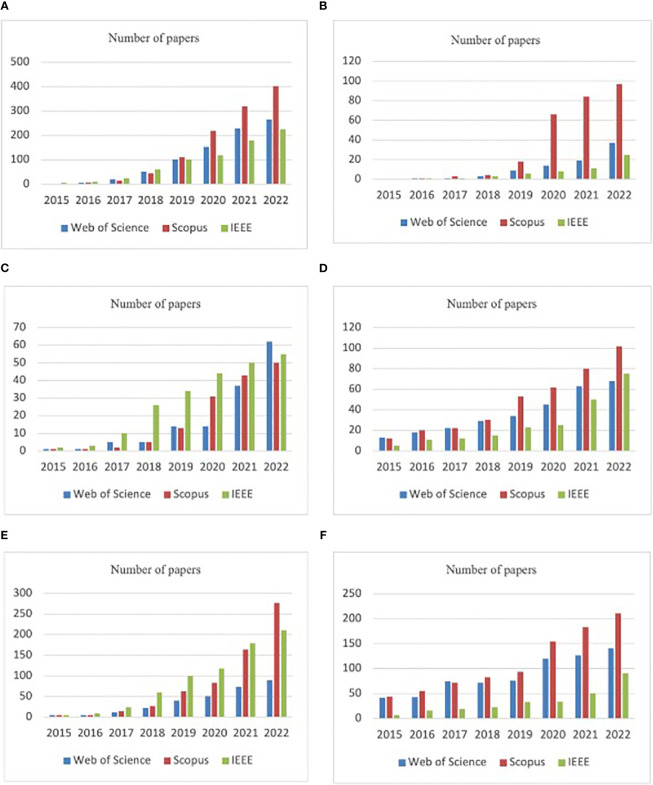
Searches for important terms in the Web of Science, Scopus, and IEEE Xplore DBs between 2015 and 2021 with AND connector: **(A)** (CNN) AND (agriculture) AND (image processing), **(B)** (CNN) AND (agriculture) AND (insects), **(C)** (CNN) AND (agriculture) AND (pest detection), **(D)** (image processing) AND (pest detection), **(E)** (CNN) AND (pest detection), **(F)** (CNN) AND (insects).

Searches for important terms and evolution as article numbers during the last years in the Web of Science, Scopus, and IEEE Xplore DBs between 2015 and 2021 with AND connector are presented in **Figure 3**: A) (CNN) AND (agriculture) AND (image processing), B) (CNN) AND (agriculture) AND (insects), C) (CNN) AND (agriculture) AND (pest detection), D) (image processing) AND (pest detection), E) (CNN) AND (pest detection), and F) (CNN) AND (insects). The graphs highlight the strong increase in the number of research articles in the connected fields in recent years regarding the use of CNN.

### 2.2 Datasets used

A robust image database (DB) is crucial for DL classification and detection because it is the foundation upon which a model is trained (Ding & Taylor, 2016). The larger and more diverse the dataset is, the better the ML model’s performance will be. A robust image dataset allows a DL model to learn a general representation of the objects or classes it is supposed to recognize. The more diverse the dataset, the better the model will be at recognizing new images that it has not seen before. Also, it enables an ML model to achieve higher accuracy in classification and detection tasks. When the dataset is comprehensive and covers a wide range of scenarios, the model can learn more accurately how to identify objects and classify them.

Insect pest databases were used in agricultural monitoring applications to track and identify the presence of insect pests that can damage crops (Turkoglu et al., 2022). These databases are typically created by agricultural organizations, universities, and research institutions that have collected data on the life cycles, presence, behavior, and distribution of various insect pests. In this regard, analyzing the papers selected for this study, several ways to construct datasets in training and validating models used for insect detection and identification were observed. Several public databases have been used by researchers in their studies to measure the performance of the implemented architectures and to test the defined models against the obtained results. **Table 1** presents a summary of the most known and frequently used databases for modern insect pest monitoring applications in agriculture.

**Table 1:** Insect DSs frequently used in agriculture applications.

DS name	Availability/Link	Classes/Observation	Number of images	Papers
IP102	Publicly/ https://github.com/xpwu95/IP102	102/Common pest species with a hierarchical taxonomy	75 222	(Ayan et al., 2020), (Butera et al., 2021), (Kasinathan et al., 2021), (Nanni et al., 2022), (Wang et al., 2022), (Wu et al., 2019)
Maryland	Publicly/ https://www.marylandbiodiversity.com/	20 600 species/ Cataloging living things	671 983	(Popescu et al., 2022)
AgriPest	Publicly/ https://github.com/liuliu66/AgriPest	14/Common pest species	49.7 K and 264.7 K annotated	(Wang et al., 2022)
Deng	Publicly/ https://doi.org/10.1016/j.biosystemseng.2018.02.008	10 species of tea plants insect pests	NA	(Deng et al., 2018) (Teng et al., 2022)
NBAIR	Publicly/ https://www.nbair.res.in/databases National Bureau of Agricultural Insect Resources	40/field crop insect images	NA	(Cardim Ferreira Lima et al., 2020) (Thenmozhi & Srinivasulu Reddy, 2019)
RGBInsect	Publicly/ http://rgbinsect.cn/	10/stored-grained insects	3757	(Li et al., 2019) (Li et al., 2020)
Xie 1	Publicly/ http://www2.ahu.edu.cn/pchen/web/insectRecognition.htm	24/field crop insect images	60 per species	(Cardim Ferreira Lima et al., 2020) (Xia et al., 2018), (Xie et al., 2015)
Xie 2	Publicly/ https://www.dlearningapp.com/web/DLFautoinsects.htm	40/field crop insect images	4500	(Cardim Ferreira Lima et al., 2020), (Ayan et al., 2020), (Nanni et al., 2022), (Xie et al., 2018)
MDP2018	Private/Multi-Class Pests Dataset 2018 https://doi.org/10.1109/ACCESS.2019.2909522	16/Insect pests	88 670	(Liu et al., 2019)
LLPD-26	Private/ https://doi.org/10.3389/fpls.2022.810546	26/insect pests	18 585	(Teng et al., 2022)
Pest24	Publicly/ http://aisys.iim.ac.cn/zhibao.html	24/field crop insect images	25 378	(Wang et al., 2020) (Wang et al., 2022)
iDigBio	Publicly/ https://www.idigbio.org/	NA/Biodiversity specimens and resources	NA	(Valan et al., 2019)
Turkey-PlantDataset	Publicly/ https://github.com/mturkoglu23/PlantDiseaseNet	15/Plant disease and pest images	4 447	(Turkoglu et al., 2022)
CPAF Dataset	Publicly/ https://drive.google.com/drive/folders/1GR4S2eqahZrLTmZlPphyfcIX5fkkV36?usp=sharing	20/insect species	73 635	(Wang et al., 2020)

Insect pest image databases often include images of insects at different life stages, including larvae and adult stages (Zhang S. et al., 2022). These images could be accompanied by additional information, such as the insect’s common name, scientific name, and the types of crops or plants that the insect pest is known to damage. This is an important aspect because the primary purpose of an insect image database is to provide a visual reference for identifying insect pests in the field. Insect pest image databases can be used as educational resources to help people learn about the different types of insect pests and their impact on agriculture and the environment (Shi et al., 2020).

One of the databases that is highlighted in the present study and that was used by the researchers in the selected papers is the IP102 DB. As presented in the acronym, it contains 102 classes of common insect pests with hierarchical taxonomy and broadly totals around 72,222 images (see **Table 1**). The database is regularly updated and maintained by a team of experts in the field of entomology. It covers a wide range of insect orders. Each entry in the IP102 Insect Database includes information on the insect’s scientific name, common name, description, habitat, diet, life cycle, behavior, and distribution, all being presented in high-quality images and illustrations, making it easy to identify different species.

The authors on IP102 DB note that existing image datasets primarily focus on everyday objects like flowers and dogs, limiting the applicability of advanced deep learning techniques in agriculture. To address this gap, they introduce a comprehensive dataset called IP102 for insect pest recognition. The authors conducted baseline experiments on the IP102 dataset using both handcrafted and deep feature-based classification methods. Their findings revealed that the dataset poses challenges related to inter-class and intra-class variance, as well as data imbalance. They anticipate that IP102 will serve as a valuable resource for future research in practical insect pest control, fine-grained visual classification, and addressing imbalanced learning challenges in this domain.

The Maryland Biodiversity Database (MBD) (Maryland Biodiversity Database, 2022) is another important database, and it has been used in various research works for the insect pest monitoring area. This database is a vast and valuable public resource that can serve as an important tool in researching various information for the insect pest area and querying it can create diverse datasets. The MBD database provides ecological information about species, including their habitats and interactions with other organisms. This can be useful for understanding the ecological context of insect pests, their host plants, and their natural predators. Its strengths lie in providing detailed species records and distribution data, facilitating ecological context for organisms, and supporting research on insect pests and native species. Researchers and conservationists benefit from its wealth of information to assess biodiversity impact and pest behavior. While not specialized in pest monitoring, MBD enhances pest management by offering a broader understanding of local ecosystems. This collaborative database stands as a crucial asset in safeguarding Maryland’s natural heritage and aiding scientific research. Scientists studying insect pests or conducting research on entomology can use the MBD to access data on insect species’ distributions and occurrences. Pest management strategies often require a comprehensive understanding of the local ecosystem. MBD can provide context by offering information on the diversity of species that may interact with or be affected by insect pests.

AgriPest introduces a domain-specific benchmark dataset for tiny wild pest detection in agriculture. This dataset contains over 49.7K images and 264.7K annotated pests, making it the largest of its kind. It aims to enhance the application of deep learning in agriculture by providing standardized data for pest detection research. AgriPest also defines sub-datasets, including challenges like pest detection and population counting, and validation subsets for various real-world scenarios. The authors build practical pest monitoring systems based on deep learning detectors and evaluate their performance using AgriPest. This dataset and associated code will be publicly available, facilitating further research in pest detection and precision agriculture.

Crafted to serve as a robust resource for training deep learning models in pest detection, Pest24 is another important DB which offers a vast repository of meticulously annotated images of agricultural pests. This paper addresses the challenges of real-time pest population monitoring in precision agriculture using AI technology. It introduces a large-scale standardized dataset called Pest24, comprising 25,378 annotated images of agricultural pests collected from automatic pest traps and imaging devices. The dataset covers 24 categories of common pests in China. On the other hand, the study applies various advanced deep learning detection methods, such as Faster RCNN, SSD, YOLOv3, and Cascade R-CNN, to detect these pests and achieves promising results for real-time field crop pest monitoring. The authors aim to advance accurate multi-pest monitoring in precision agriculture and provide a valuable object detection benchmark for the machine vision community.

The analysis of Pest24 highlights three key factors influencing pest detection accuracy: relative scale, number of instances, and object adhesion. Due to the scarcity of multi-target pest image big data, Pest24 holds great importance as a resource for advancing intelligent field crop pest monitoring. Characterized by its large-scale data, small relative object scales, high object similarity, and dense distribution, Pest24 presents unique challenges for deep learning-based object detection methods and is poised to drive progress in pest detection for precision agriculture while serving as a specialized benchmark for the computer vision community. Beyond its application in precision agriculture, Pest24 serves as an invaluable benchmark for the machine vision community, fostering advancements in specialized object detection. Future work aims to expand the dataset with more diverse multi-pest images from various practical.

Xie1 and Xie2 databases were other important resources in creating databases or testing and training the architectures defined in various works (**Table 1**). Because these datasets are not large some authors have often resorted to augmentation techniques to increase the size of these datasets. The Xie2 dataset also called D0 contains 40 classes of insect pests represented in 4508 RGB images of 200 x 200px resolution.

Although there are several public databases illustrating and grouping various common classes of insect pests, most of the authors used their own datasets in solving the problems specific (Bhoi et al., 2021; Rajeena et al., 2022). Creating proprietary databases for insect pest detection or monitoring using NNs can help improve the accuracy and specificity of pest detection systems, while also providing flexibility and cost-effectiveness (Segalla et al., 2020; Hong et al., 2021). From the point of view of flexibility, creating its own database offers absolute control of the data that is attached to train the NNs for insect pest monitoring. This is about how the data set can be adjusted as needed to meet the changing need for insect pest families and environmental changes that may occur rapidly. Complete control of the specificity of pest populations was discussed in several works to describe the specificity zone (Khanramaki et al., 2021). By creating proprietary databases, specific insect pests can be tailored and described regarding each context and interest in pest recognition and monitoring. This can help ensure that the NNs are able to accurately identify and differentiate between the specific insect pests, rather than simply providing a general detection of any insect in the image (Liu and Wang, 2020; Xu et al., 2022).

It is very important that the data set describes a real context to solve real problems with increased accuracy. What was observed in this regard as part of the present study in relation to the performances obtained by the authors in various works was a tendency to create robust datasets in increasing performances. The larger the database used for training and validating NNs, the higher the accuracy of the created models can be (Knyshov et al., 2021; Liu et al., 2022). The database used is determined by the precise study objectives and the sort of data required. Researchers interested in insect pest recognition, for example, may pick IP102 or Pest24, but those needing ecological context may prefer MBD. AgriPest is appropriate for precision agricultural research. Collectively, these databases help to advance pest detection and agricultural research. When paired with these different datasets, CNNs provide a very effective tool for insect pest study and control. They can help to increase pest detection accuracy, understand pest behavior in ecological contexts, and improve real-time monitoring and control tactics in precision agriculture. Researchers and practitioners may use these datasets to create more effective and efficient pest-related solutions in agriculture.

In another scenario, from a cost point of view, creating own database can be a cost-effective alternative. Test and training data creation solutions can capture data using low-cost methods like phones or digital cameras, which is a pretty good starting point. Where the data set is acquired using drones, high-fidelity cameras, robots, or specialized human resources, the cost of acquiring and creating the reference data set for pest monitoring can increase commensurately with the size and quality of data acquired (Xing et al., 2019; Tian H. et al., 2020; Genaev et al., 2022).

The organization of the data set represents another aspect noted by the authors in the development of models for harmful insect and pest detection in modern agriculture. In general, for training and evaluation using CNNs for pest detection and identification, the dataset division commonly includes training and validation sets or training, validation, and testing sets. The most common ratio observed in the last split was 70% for training, 20% for validation, and 10% for testing (Huang et al., 2022). The other ratio could include 80% for training and 20% for testing (Du et al., 2022; Zhang S. et al., 2022), or 70% with 30% respectively (Ahmad et al., 2022).

Regarding the dataset, the authors also followed techniques like data augmentation (Du et al., 2022; Zhang et al., 2023). Data augmentation in the context of CNNs is the process of producing additional training examples by applying various changes to existing pictures in the training dataset (Albanese et al., 2021). Geometric changes such as random rotation, horizontal and vertical flips, random cropping, and transformations such as brightness modifications or color jitter are examples of frequent transformations used for data augmentation in CNNs (Padmanabhuni and Gera, 2022). Adding random cropping can assist the model in learning to distinguish things that are not centered in the image (Genaev et al., 2022).

For data augmentation, some of the new trends include synthetic data generation to increase the number of samples if the number of representatives of a class is insufficient (Abbas et al., 2021). Using generative models to create synthetic images is one novel method of data augmentation. Augmentation through synthetic data generation is a novel technique of generating new training data using computer algorithms rather than gathering real-world data (Huang et al., 2022). The purpose of this method is to enhance the quantity and variety of the dataset, which can improve the performance of ML models (Divyanth et al., 2022). Synthetic data generation could address issues such as imbalanced datasets, lack of data privacy, and limited data availability (Lu et al., 2019). For the topic of agricultural pests, this can be done in a variety of ways. There are several methods for creating synthetic data for CNNs (Karam et al., 2022), including generative adversarial networks (GANs), deep learning picture synthesis, data augmentation, and data interpolation (Padmanabhuni and Gera, 2022). Conditional GAN was used by (Abbas et al., 2021) to generate synthetic images for tomato pests and to improve the performances. Another performance improvement was noted by (Divyanth et al., 2022) by creating an artificially generated dataset using GAN.

For the testing phase, the acquisition of digital images from real contexts can be noted. This was pursued by the authors to test the NN architectures they created and optimized against the real contexts, using pest images in the field (Brunelli et al., 2020). For modern agriculture, there are some ways of acquiring digital images, using various systems and techniques (Terentev et al., 2022). The present study identified four important directions that describe image acquisition vectors and were grouped and described in **Table 2**. Based on the analyzed references, the performances obtained using the created databases were also noted. In this sense, satisfactory results are observed, and at the same time, it is important to note that the methods of image acquisition are done in an optimized framework and represent a strong point attached to the research areas in this field. For the modern agricultural area, the acquisition of data for the creation of models and automatic solutions in pest monitoring represents an extensive process that can include several resources (Nanni et al., 2022).

**Table 2:** Modality of image acquisition.

Image acquisition vector	Agricultural crop/images	Performances	Papers
Human operators (with camera or smartphone)	Oil palm/8000 Eggplant/NA NA/563 Fruits/365	ACC: 89% R^2 = ^0.85 to 0.95 ACC: 94.3% F1 Score: 83.8%	(Ahmad et al., 2021) (Bereciartua-Pérez et al., 2022) (Cochero et al., 2022) (Genaev et al., 2022)
Pheromone-based traps and cameras	Apple orchard/8000 Apple/300 Vegetables/1789 Forest/50 Greenhouse/400	ACC: 97.9% training ACC: 97% training, 93% validation F1 Score: 83.8% ACC: 95.3% - 97.89% F1 Score: 90% - 92%	(Albanese et al., 2021) (Brunelli et al., 2020) (Guo et al., 2021) (Hong et al., 2021) (Rustia et al., 2020)
UAV	Forest/4710 Rice/NA Weeds, Potato, Grapes/600 NA/500 Maize/5691 Eucalyptus/4930	PRE: 70% ACC: 80% ACC: 90% PRE: 85%, F1 Score: 55% ACC: 97.59% - 98.77% ACC: 98.45%	(Aota et al., 2021) (Bhoi et al., 2021) (Bouroubi et al., 2018) (De Cesaro Júnior et al., 2022) (Dai et al., 2021) (Dos Santos et al., 2022)
Terrestrial vehicles and camera	Pomelo orchard/510	ACC: 95.83%	(Partel et al., 2019) (Tian G. et al., 2020)

**Table 2** summarizes the most common data gathering methods, with UAVs and pheromone traps emerging as the most popular options. This section will evaluate the benefits and drawbacks of different techniques. The integration of ML and deep learning DL for automated data processing, with a special focus on remote sensing and sensory data for complete area mapping, is an emerging research field. It is worth mentioning that remote sensing, as investigated by (Stefas et al., 2016; Ahmad et al., 2021), has several applications in fields such as agriculture and forestry.

Unmanned Aerial Vehicles (UAVs) are gaining remarkable traction across diverse domains, with agriculture and environmental monitoring being prominent beneficiaries. One of their vital applications lies in the realm of pest detection and management within agricultural crops Mu et al., 2018. UAVs offer versatile data acquisition methods, including high-resolution imagery and sensory capabilities (Tian H. et al., 2020; Cochero et al., 2022). Equipped with high-resolution cameras, UAVs excel at capturing images and videos of crops, facilitating the identification of insect pests (Tian H. et al., 2020; Cochero et al., 2022). Subsequently, these images can undergo automated pest detection using ML algorithms (Preti et al., 2021). Moreover, UAVs can be equipped with sensors for detecting specific chemicals in the air or on plant surfaces, thus enabling pest identification, as well as treatment efficacy monitoring (Velusamy et al., 2022). To combat identified pests, certain UAVs are equipped with precision sprayers, targeting affected areas with minimal chemical usage and environmental impact (Iost Filho et al., 2019; Li C. et al., 2022). Thermal cameras mounted on UAVs provide valuable temperature data, aiding in pinpointing stressed or pest-infested crop areas due to temperature differences (Yuan & Choi, 2021). UAVs also use multispectral cameras, such as infrared and hyperspectral imaging, in addition to typical RGB images, which considerably improves the accuracy of pest detection models (Terentev et al., 2022). Another current technique employs lidar sensors to collect high-resolution 3D pictures of agricultural fields, allowing for the identification of pest-infested areas (Dong et al., 2018; López-Granados et al., 2019). Lidar imaging also provides information about crop dimensions, growth patterns, and prospective yield (Johansen et al, 2018; Ampatzidis et al., 2020).

Nonetheless, there are several drawbacks to the UAV-based strategy. UAVs, in general, have limited payload capacity, restricting their ability to carry large amounts of equipment and sensors. Furthermore, UAV flight durations are limited, often ranging from 20 to 30 minutes depending on the type and payload. As a result, covering large regions may demand numerous flights, which can be both time-consuming and costly (Dong et al., 2020). While UAVs excel in collecting high-resolution photographs of crops and insect pests, image analysis algorithms’ accuracy may be limited, necessitating professional analysis. Furthermore, the use of UAVs for data collecting is vulnerable to weather and legal limitations. These variables might limit the capacity to collect insect pest data during certain seasons or geographical locations. Many nations have tight UAV laws that include flying limitations as well as criteria for permissible equipment and sensors (Csillik et al., 2018).

Another way of data acquisition for insect monitoring in modern agriculture is based on pheromone traps (**Table 1**). Data acquisition using pheromone traps is a useful tool for monitoring and controlling insect pests in agriculture and forestry. Pheromone traps are placed in strategic locations throughout a crop. The number and placement depend on the type of insect pest being targeted and the size of the area being monitored. Pheromone traps need to be checked regularly to ensure that they are working properly. Digital cameras can be attached to the pheromone traps to capture images of the trapped insects. The traps should be monitored regularly, typically every 1-2 weeks. During each monitoring visit, the traps are checked for trapped insects, and the digital cameras are checked to ensure they are functioning correctly.

While pheromone traps can be an effective tool for monitoring and managing insect pests, there are some disadvantages to their use. They are only effective against insect pests that are attracted to specific pheromones (Cardim Ferreira Lima et al., 2020). On the other hand, their effectiveness is limited to the area in which they are placed (Toscano-Miranda et al., 2022). Pheromone traps can attract not only the target insect pest but also non-target species that are attracted to the same pheromones. Additionally, pheromone traps can give an incomplete or inaccurate representation of the population of insect pests. This is because some individuals of the pest species may not respond to the pheromone lure or may be located outside the trapping area. This can lead to incorrect decisions about pest management strategies.

In agriculture, insect pest identification and monitoring are key parts of precision farming because they have a direct influence on crop production, quality, and overall agricultural sustainability. CNNs, in conjunction with specialist databases, provide significant promise for tackling the issues connected with insect pest control. Deep learning architectures, object identification, categorization and taxonomy, and real-time monitoring may be essential elements of neural networks applied to these datasets, as demonstrated in the selected research. With the use of CNNs and specialized datasets, insect pest identification and monitoring have entered a new age. This synergy has the potential to lead to more accurate, timely, and environmentally conscious pest management solutions. Continuous research, multidisciplinary cooperation, and an emphasis on practical application are required to fully achieve this promise. As technology advances, the future of insect pest identification and monitoring in agriculture remains bright, with the potential to greatly contribute to global food security and sustainable agriculture practices.

### 2.3 Neural networks used in insect detection, segmentation, and classification

**Figure 4:**
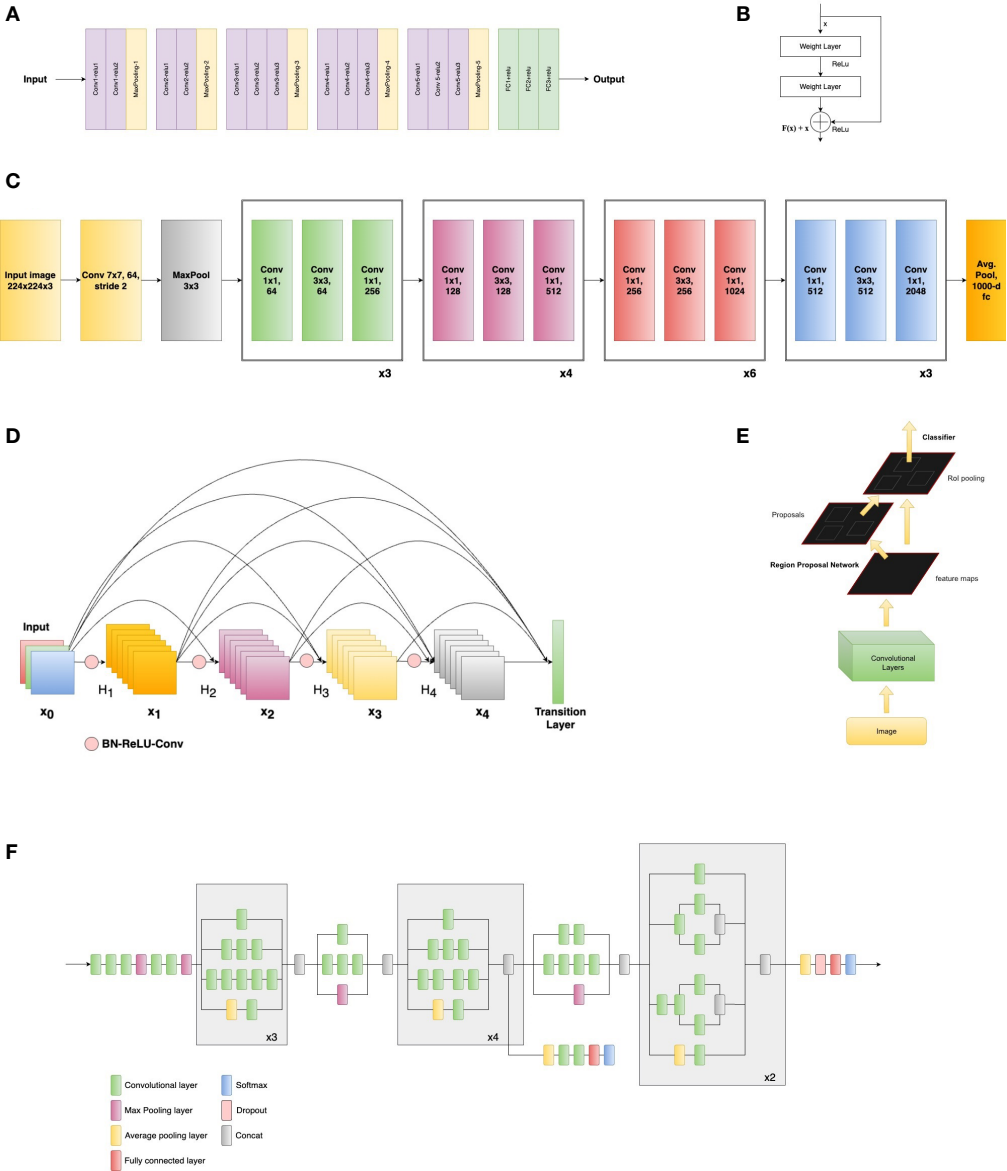
Examples of neural networks used: **(A)** VGG-16 architecture (adapted from Simonyan and Zisserman 2014, **(B)** Residual block example (adapted from He et al., 2016), **(C)** Example architecture of ResNet-50 (adapted from He et al., 2016), **(D)** Dense block example (adapted from Huang et al., 2017), **(E)** Faster RCNN architecture (adapted from Ren et al., 2015), **(F)** Inception V3 architecture (adapted from Szegedy et al., 2016).

Automated monitoring systems use sensors and cameras to detect and identify insect pests (Amorim et al., 2019). These systems can be connected to the internet, allowing farmers to receive real-time information about pest populations. There are many solutions and methodologies based on image processing, DL, and NNs. CNNs are particularly well suited for tasks involving the detection of small objects, such as insects, within an image. In this scenario, CNNs are a powerful tool for pest detection and have been shown to achieve high accuracy in many applications. One key advantage of CNNs for pest detection is their ability to handle complex images. For example, a CNN can be trained to detect pests in images that contain multiple objects, different backgrounds, and varying lighting conditions (Fang et al., 2020). Additionally, CNNs can be trained on a large dataset of images, which can help improve the accuracy of the model. Another advantage of CNNs for monitoring crops for pest detection is their real-time ability (Ayan et al., 2020). On the other hand, one of the most significant advancements in this field is the development of transfer learning, where a pre-trained CNN model is fine-tuned on a smaller dataset of pest images. Some of the most used NNs for insect and pest detection and classification are presented in **Figure 4**.

VGG Net (Visual Geometry Group) (Simonyan & Zisserman, 2014) is a key architecture used in insect detection and monitoring, especially VGG-16 and VGG-19. The architecture is widely used in computer vision applications such as object detection and image segmentation (Popescu et al., 2022). The architecture for VGG-16 (Ramadhan & Baykara, 2022) is shown in **Figure 4A**, and it was the most used for insect detection and classification tasks. The convolutional layers are responsible for extracting features from the input image, while the pooling layers reduce the spatial dimensions of the feature maps to reduce computation time. The fully connected layers are used to classify the features extracted by the convolutional and pooling layers. The most used, VGG-16 model has a total of 16 layers and the VGG-19 has 19 layers being a modified version of VGG-16 with the addition of the new three convolutional layers.

Residual Network (ResNet) is another CNN family used in insect monitoring for modern agriculture. ResNet-18, ResNet-34, ResNet-50, ResNet-101, and ResNet-152 are variants of the CNN architecture that was introduced in 2015 by researchers at Microsoft (He et al., 2016). The key innovation of ResNet is the use of “residual connections,” or shortcut connections, that allow the network to learn identity mapping and make it easier to train very deep networks. This is shown in **Figure 4B** as a residual block example. According to the investigated papers, ResNet-50 was the most used for insect detection and classification tasks, and the basic architecture is shown in **Figure 4C**.

The R-CNN (Region-based CNN) architecture is a type of object detection model that uses a combination of CNNs and region proposal algorithms to detect objects within an image (Ren et al., 2015) and was also used for insect monitoring. It is a two-stage process that first generates a set of region proposals and then uses a CNN to classify and refine the proposals. The first stage of the R-CNN architecture is the region proposal algorithm, which generates a set of regions or “proposals” that may contain an object of interest. These regions are then passed to the second stage of the R-CNN architecture, which is the CNN. This is used to classify and refine the regions generated by the region proposal algorithm and it is done by extracting features from each region and passing them through a series of convolutional and fully connected layers. In this context, another architecture often used for insect detection tasks was Faster R-CNN. This is a type of object detection model that uses a CNN to extract features from an image and then uses a region proposal network (RPN) to propose regions that may contain objects. The feature extractor typically uses a pre-trained CNN, such as VGG or ResNet, to extract features from the input image. The main advantage of Faster R-CNN over other object detection models is its efficiency, as it shares computation between the RPN and the classifier. The Faster R-CNN architecture, adapted from (Ren et al., 2015) is presented in **Figure 4E**.

The Inception CNN architecture (Szegedy et al., 2015) is also representative of insect classification and detection. This deep CNN architecture utilizes a combination of convolutional, pooling, and inception modules to efficiently learn hierarchical representations of visual data. The novel aspect is that it includes a series of components named Inception modules that apply a combination of convolutional and pooling layers at different scales, allowing the network to efficiently capture and learn both the high-level and low-level features of the image. This review highlighted that the most used architecture from this family, for insect detection and classification was the InceptionV3 (Szegedy et al., 2016). Following the structure and features presented previously, the basic scheme of Inception V3 can be viewed in **Figure 4F** (adapted from Szegedy et al., 2016).

Dense Convolutional Network (DenseNet) is another CNN family used for insect detection and classification. This neural network architecture is characterized by dense layers Huang et al., 2020. Each layer is connected to every other layer in the network (Huang et al., 2017). This creates a dense network of connections, which allows for a more efficient flow of information and a greater capacity for learning. A dense block is shown in **Figure 4D**, adapted from (Huang et al., 2017). One of the main advantages of DenseNet architecture is its ability to effectively handle large amounts of data and complex patterns (Huang et al., 2017).

YOLO (You Only Look Once) is another state-of-the-art family that is widely used in the modern agricultural sector for real-time insect detection and monitoring. YOLO (Redmon et al., 2016) is an object detection algorithm that uses a single stage to perform object detection. Unlike other object detection algorithms that rely on region proposals, YOLO uses a grid of cells to divide the image into smaller regions and predicts the object class and location for each cell. The algorithm is trained on large datasets, such as the COCO (Common Objects in Context) or ImageNet, and has been designed to be fast and accurate. Different variants from this family were used: YOLOv2 (Redmon & Farhadi, 2017), YOLOv3 (Redmon & Farhadi, 2018), YOLOv4 (Bochkovskiy et al., 2020), YOLOv5s, YOLOv5m, and YOLOv5l (Ultralytics, 2020).

**Figure 5:**
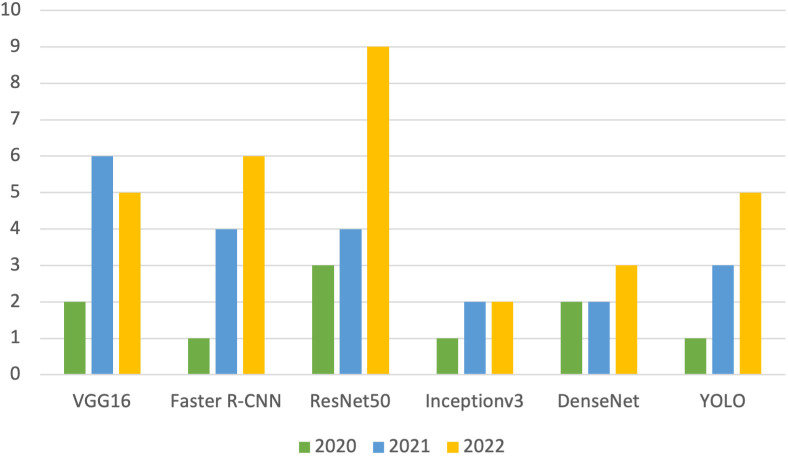
The graph of the evolution in the last three years of the most used neural networks.

A synthetic presentation of NNs used for insect and pest detection and classification in agricultural applications is given in **Table 3**. Based on the information from **Table 3**, the graph in **Figure 5** describes the evolution over the last three years of the most used neural networks for insect monitoring in modern agriculture.

**Table 3:** CNN used in insect and pest detection.

CNN family/References	Representatives/configuration	Function	Performances	Papers
AlexNet 5	AlexNet	Classification	ACC: 80.3% - 91.31.%, F1 score: 96%	[(Khanramaki et al., 2021), (Li et al., 2019), (Malathi and Gopinath, 2021), (Xu et al., 2022), (Divyanth et al., 2022)
CapsNet 2	CapsNet/modified	Classification	ACC: 82.4%, PRE: 75.41%	(Xu et al., 2022), (Zhang S. et al., 2022)
CNN 8	CNN	Classification	ACC: 91.5% - 98,6% F1 score: 95%	(Chodey & Shariff, 2021), (Hossain et al., 2019), (Espinoza et al., 2016), (Kasinathan et al., 2021), (Sharma et al., 2020), (Singh et al., 2021)
	BPNN	Classification	ACC: 91%	(Zhu et al., 2020)
DenseNet 8	DenseNet 121	Detection and classification	ACC: 88.06% - 99.1%	(Abbas et al., 2021), (Sanghavi et al., 2022), (Zhang & Chen, 2020), (Shi et al., 2020)
	DenseNet 169	Detection	mAP: 92.3%	(Butera et al., 2021)
	DenseNet 201	Detection and classification	ACC: 79.01%, 95.52%	(Nanni et al., 2022), (Singh et al., 2021)
	Weakly DenseNet-16	Classification	ACC: 93.42%	(Xing et al., 2019)
EfficientNet 4	EfficientNet	Detection	ACC: 97.89% - 99.1%	(Dai et al., 2021), (Sanghavi et al., 2022), (Takimoto et al., 2021)
	EfficientNet B0	Detection	ACC: 94.25%	(Nanni et al., 2022)
EfficientDet 1	EfficientDet D0	Detection	ACC: 95.3% - 97.9%	(Hong et al., 2021)
GoogLeNet 1	GoogLeNet with Inception modules	Classification	ACC: 91.02%	(Malathi and Gopinath, 2021)
Inception 10	Inception v3	Classification	ACC: 75.3% - 99.04% mAP: 71%	(Ayan et al., 2020), (Fang et al., 2020), (Hansen et al., 2019), (Rajeena et al., 2022), (Sanghavi et al., 2022), (Singh et al., 2021), (Wang et al., 2020), (Liu et al., 2022)
	Inception ResNetv2	Detection	ACC: 91.14%	(Khanramaki et al., 2021), (Singh et al., 2021)
LeNet 3	LeNet5	Classification	ACC: 93.1% - 96.1%, PRE: 94%	(Albanese et al., 2021), (Ding & Taylor, 2016) (Segalla et al., 2020)
MobileNet 10	MobileNet	Detection and classification	ACC: 82.10% - 97.39%	(Ayan et al., 2020), (Singh et al., 2021), (Xing et al., 2019)
	MobileNetv2	Detection	ACC: 81.32% - 96.29%	(Albanese et al., 2021), (Hong et al., 2021), (Nanni et al., 2022), (Rajeena et al., 2022), (Xing et al., 2019), (Zhang & Chen, 2020)
	MobileNetv3	Detection	mAP: 92.66%	(Butera et al., 2021)
	Optimized MobileNet	Classification	ACC: 95.04%	(Rimal et al., 2022)
NASNet/1	NASNetMobile	Classification	ACC: 73.46%	(Singh et al., 2021)
Perceptron/1	Multi-layer perceptron	Detection	ACC: 98.45%	(Dos Santos et al., 2022)
R-CNN/13	Cascade R-CNN	Detection	mAP: 70.83%	(Dos Santos et al., 2022)
	Faster R-CNN	Detection and classification	ACC: 60,2% - 99% F1: 85.5% - 99.5% mAP: 65.58% - 89.1%	(Ahmad et al., 2021), (Alsanea et al., 2022), (Butera et al., 2021), (Du et al., 2022), (Guo et al., 2021), (Hong et al., 2021), (Li et al., 2019), (Liu et al., 2019), (Wang et al., 2022), (Shi et al., 2020)
	Mask R-CNN	Detection and segmentation	PRE: 85%	(De Cesaro Júnior et al., 2022)
	MSR-RCNN/ResNet-50 backbone	Detection	mAP: 67.4%	(Teng et al., 2022)
RegNet 1	RegNet	Detection	ACC: 98.07%	(Dai et al., 2021)
ResNet 31	ResNet/modified	Detection	ACC: 95.83%	(Tian G. et al., 2020),
	ResNet 18/modified	Detection	ACC: 60.3%	(Roosjen et al., 2020)
	ResNet 34	Detection	ACC: 94.3%, 91.2%	(Cochero et al., 2022), (Malathi and Gopinath, 2021)
	ResNet 50	Classification	ACC: 43.99% - 99.04% F1 score: 55% - 92.6% mAP: 74,24% - 88.5%	(Ayan et al., 2020), (Bereciartua-Pérez et al., 2022), (Butera et al., 2021), (De Cesaro Júnior et al., 2022), (Fang et al., 2020), (Dai et al., 2021), (Khanramaki et al., 2021), (Li et al., 2019), (Liu et al., 2019), (Liu et al., 2022), (Malathi and Gopinath, 2021), (Nanni et al., 2022), (Rajeena et al., 2022), (Sanghavi et al., 2022), (Wang et al., 2020), (Wang et al., 2022), (Xu et al., 2022)
	ResNet 53	Detection	mAP: 77.29%	(Lv et al., 2022)
	ResNet 101	Detection	mAP: 85.53% - 99.5%	(Hong et al., 2021), (Li et al., 2019), (Liu et al., 2019), (Lv et al., 2022), (Wang et al., 2022), (Zhang & Chen, 2020), (Shi et al., 2020)
	ResNet 152	Detection	ACC: 96.31%	(Zhang & Chen, 2020)
	ResNeXt-50	Classification	ACC: 86.5%	(Li C. et al., 2022)
RetinaNet 3	RetinaNet	Detection	mAP: 65.03% - 94.77%	(Li et al., 2020), (Wang et al., 2022)
	RetinaNet50	Detection	mAP: 86.40%	(Hong et al., 2021)
SqueezeNet 1	SqueezeNet	Classification	ACC: 94.02%	(Ayan et al., 2020)
ShuffleNet 2	ShuffleNet v1	Classification	ACC: 83.58%	(Xing et al., 2019)
	ShuffleNet v2	Classification	ACC: 83.58%	(Xing et al., 2019)
SSD 3	SSD	Detection	PRE: 70%	(Aota et al., 2021)
	SSD with MobileNetv2	Detection	mAP: 84.54%	(Hong et al., 2021)
	SSD/with VGG-16 and ResNet-50	Detection	mAP: 63.38%	(Wang et al., 2022)
VGG 23	VGG16/modified	Classification	ACC: 67% - 97.9% R^2 = ^0.85 to 0.95	(Albanese et al., 2021), (Ayan et al., 2020), (Bereciartua-Pérez et al., 2022), (Khanramaki et al., 2021), (Knyshov et al., 2021), (Kusrini et al., 2021), (Li et al., 2019), (Nazri et al., 2018), (Rajeena et al., 2022), (Sanghavi et al., 2022), (Singh et al., 2021), (Valan et al., 2019), (Wang et al., 2020), (Wu et al., 2019), (Xing et al., 2019), (Zhang S. et al., 2022)
	VGG16/modified	Detection	F1:95.25%, mAP: 78.20%, PRE: 99%	(Segalla et al., 2020), (Wang et al., 2022), (Shi et al., 2020)
	VGG19	Classification	ACC: 74.07% - 99.02%	(Ayan et al., 2020), (Fang et al., 2020), (Rajeena et al., 2022), (Singh et al., 2021)
	VGG19/improved + RPN	Detection	mAP: 89.22%	(Xia et al., 2018)
Xception 4	Xception	Classification	ACC: 74.07% - 97.98 PRE: 77%	(Ayan et al., 2020), (Fang et al., 2020), (Rajeena et al., 2022), (Singh et al., 2021), (Kuzuhara et al., 2020)
YOLO 14	YOLO	Detection	ACC: 88.06% - 92.50%	(Shi et al., 2020), (Zhong et al., 2018)
	YOLOv3/improved	Detection	PRE: 77%, mAP: 77.29%, F1: 87% - 90%	(Kuzuhara et al., 2020), (Liu and Wang, 2020) (Lv et al., 2022), (Partel et al., 2019), (Rustia et al., 2020)
	Tiny-YOLOv3	Detection	F1 Score: 90% - 92%	(Rustia et al., 2020)
	YOLOv4	Detection	F1 Score: 55% - 83.8%	(Genaev et al., 2022), (Takimoto et al., 2021)
	YOLOv5	Detection	ACC: 98.45%, mAP: 77.0% -99.2%	(Bereciartua-Pérez et al., 2022), (Dos Santos et al., 2022), (Zhang Y. et al., 2022), (Zhang et al., 2023)
ZF Net 2	ZF Net	Detection	mAP: 88.5%, 75.46%	(Li et al., 2019), (Liu et al., 2019)

This study primarily centers its focus on exploring emerging trends in CNNs for insect pest detection and monitoring through the innovative application of new combinations, while also acknowledging classic CNN models as reference points. This approach aligns with the prevalent practice in the field, where most studies strive to strike a balance between pioneering CNN architectures and established, foundational models. This dual perspective, embracing innovation while respecting tradition, mirrors a common practice observed in the contemporary studies within the deep learning community. Researchers understand that leveraging the strengths of both new and classic CNN models can yield comprehensive insights and solutions, ultimately driving the field forward.

Regarding key trends and advancements, CNNs continued to be a popular choice for image-based insect pest detection. Researchers were developing and fine-tuning CNN architectures to achieve higher accuracy in recognizing and classifying pests from images. Transfer learning techniques were becoming increasingly important in this domain. Researchers were pre-training CNN models on large datasets and then fine-tuning them for insect pest detection tasks. This approach helped in achieving better results even with limited labeled data for specific pests. For object detection and localization, object detection models like Faster R-CNN, YOLO and SSD were adapted for insect pest monitoring. These models not only classified pests but also provided bounding box coordinates, which is crucial for precise pest localization. As a new trend, researchers were experimenting with advanced data augmentation techniques to improve model robustness. Techniques like GANs were used to create synthetic pest images to augment the training dataset. Next, focusing on network architectures, capsule networks, which aim to address the limitations of traditional CNNs in handling hierarchical features, have been explored for insect pest recognition (Xu et al., 2022; Zhang S. et al., 2022). They can capture the spatial hierarchies of pest body parts for improved classification. Some researchers have proposed hybrid architectures that combine the strengths of CNNs for image processing and recurrent neural networks (RNNs) for sequential data processing. This is particularly useful when tracking pests’ movements over time (Butera et al., 2021; Alsanea et al., 2022; Du et al., 2022). To make pest detection systems more transparent and interpretable, explainable AI in architectures techniques have been integrated into neural network architectures. This allows users to understand why a particular pest detection decision was made. Researchers often choose or design architectures based on the unique characteristics and challenges of the pests they are targeting and the monitoring environment. Advancements in neural network architectures for insect pest detection and monitoring are ongoing, so staying up to date with the latest research papers and developments in the field is essential for the most current insights.

### 2.4 Performance indicators

Looking at the area of impact and innovation, the new trends stand out with high-performance indices in relation to the area of pest identification. Attaching these was done to create a comparison area. Since the research was based on deep learning models, the indicators most used as evaluation methods of these models were highlighted as part of this study, being represented by accuracy, precision, sensitivity, specificity, F1 score, Jaccard index, mean average precision (mAP), and sometimes R2. Names and calculation formulas are attached in **Table 4**. The most used performance indicators were mAP, accuracy, and F1 score. Representative indices were also extracted from the creation of the confusion matrix (Ahmad et al., 2022) where the values for TP – True Positive, TN – True Negative, FP – False Positive, and FN – False Negative are indicated.

**Table 4:** Performance indicators used in the review.

Indicator	Formula	Indicator	Formula
Accuracy (ACC)	$ACC=\frac{TP+TN}{TP+TN+FP+FN}$	Sensitivity (SEN)	$SEN=\frac{TP}{TP+FN}$
Precision (PRE)	$PRE=\;\frac{TP}{TP+FP}$	Specificity (SPE)	$SPE=\;\frac{TN}{TN+FP}$
F1 Score (F1)	$F1=\;\frac{2\cdot TP}{2\cdot TP+FP+FN}$	Jaccard index (j)	$j=\;\frac{TP}{TP+FN+FP}$
Mean Average Precision (mAP)	$mAP=\;\frac{1}{N}*\;\sum_{i=1}^{N}AP_{i}$	R^2^	$R^{2}\left(y,ŷ\right)=\;1-\;\frac{\sum_{i=1}^{n}{\left(y_{i}-{ŷ}_{i}\right)}^{2}}{\sum_{i=1}^{n}{\left(y_{i}-ӯ\right)}^{2}}$ $R^{2}=\;\frac{Explained\;variation}{Total\;Variation}$

### 2.5 Software used

This study underlines the need of tracking the software used in NNs (**Table 5**). This is especially important given the fast developments in NNs and the advent of new software and approaches. Nevertheless, various software programs may yield somewhat different results due to differences in implementation and optimization strategies. Knowing what software was used allows others to replicate and validate the results. This is especially significant for improving the area and expanding on previous studies. Furthermore, knowing the software utilized helps enhance collaboration in the fields of NNs and PA. It allows academics to share code and data, enabling the flow of ideas and speeding up research and development.

**Table 5:** Software used.

Software	Description	Link	Papers
PyTorch	▪ An open-source machine learning framework ▪ Based on Python programming language and Torch library	https://pytorch.org/	(Cochero et al., 2022), (Du et al., 2022), (Dai et al., 2021), (Guo et al., 2021), (Huang et al., 2022), (Lv et al., 2022), (Wang et al., 2022), (Zhang Y. et al., 2022), (Zhang et al., 2023), (Shi et al., 2020)
TensorFlow	▪ An end-to-end open-source machine learning platform	https://www.tensorflow.org/	(Ahmad et al., 2021), (Alsanea et al., 2022), (Bereciartua-Pérez et al., 2022), (De Cesaro Júnior et al., 2022), (Fang et al., 2020), (Guo et al., 2021), (Hossain et al., 2019), (Karam et al., 2022), (Knyshov et al., 2021), (Rajeena et al., 2022), (Rimal et al., 2022), (Sharma et al., 2020), (Takimoto et al., 2021), (Valan et al., 2019), (Wang et al., 2020), (Wang et al., 2022), (Wu et al., 2019)
Keras	▪ High-level, modular, and flexible open-source neural network library and API based on Python programming language	https://keras.io/	(Ayan et al., 2020), (Fang et al., 2020), (Hossain et al., 2019), (Karam et al., 2022), (Knyshov et al., 2021), (Lu et al., 2019)
Imagga Cloud API	▪ Image recognition API as a service	https://imagga.com /	(Bhoi et al., 2021)
Fastai	▪ Deep learning library	https://www.fast.ai/	(Cochero et al., 2022)
MathWorks Matlab	▪ Programming and numeric platform designed for engineers and scientists	https://www.mathworks.com/products/matlab.html	(Divyanth et al., 2022), (Nagar and Sharma, 2021)

As can be observed from **Table 5**, Tensorflow in combination with Keras is the most popular choice for software development in pest detection or identification systems using CNNs (Fang et al., 2020). The second popular way of software implementation, showing increasingly high and modern adoption, is represented by PyTorch with the attached torch and torch-vision libraries.

TensorFlow is an open-source software library developed by Google for building and training ML models (Abadi et al., 2016). It is a popular and powerful DL framework that provides a wide range of tools and APIs for building and training models (Wang et al., 2022). TensorFlow is a library for numerical computation that is particularly well-suited to the computation of large-scale linear algebra operations, which are a common component of many ML algorithms. It provides a wide range of tools for building and training DL models, including CNNs and recurrent NNs. It also includes support for distributed training and deployment on different hardware platforms. For the task of detection of harmful insects and pests in modern agriculture, it was a popular choice (**Table 5**).

Keras is an open-source software library written in Python that provides a high-level interface for building and training DL models (Chollet et al., 2015). It is built on top of other popular DL frameworks, including TensorFlow, and provides a simple and intuitive API for defining and training models. Keras was designed with the goal of making DL accessible to a wider audience, including researchers, students, and developers with limited ML experience.

For insect monitoring tasks, another software used was PyTorch. Based on the analyzed papers, a strong adoption of the framework in pest detection tasks is observed in the last three years, especially in 2022. Facebook (actual Meta) team created PyTorch as an open-source machine learning framework (Paszke et al., 2019). It is a well-known and sophisticated DL framework that offers a variety of tools and APIs for developing and training ML models. Torch is a scientific computing framework that enables efficient tensor operations and automated differentiation. PyTorch is built on top of the Torch library and improves these capabilities by including a dynamic computational graph, allowing for more flexible and intuitive model creation, and debugging. PyTorch includes a variety of tools and APIs for developing and training DL models such as CNNs, recurrent NNs, and others.

The MATLAB programming and numerical computing platform do not have the same characteristics as the libraries and deep learning frameworks like Tensorflow + Keras or PyTorch, based on the performance and flexibility associated with the Python programming language in which they are implemented. MATLAB (matrix laboratory) is a programming environment and a programming language used primarily for numerical computing and scientific computing (MathWorks Matlab 22). MathWorks MATLAB provides a wide range of built-in functions and tools specifically designed for image processing and computer vision applications (Nagar and Sharma, 2021). MATLAB’s Image Processing Toolbox provides a comprehensive set of tools for image analysis, filtering, segmentation, feature extraction, and object recognition (Divyanth et al., 2022). The toolbox includes functions for common image processing tasks such as image smoothing, noise reduction, edge detection, and morphological operations. MATLAB also provides support for deep learning and machine learning, which can be used for image classification and object recognition tasks.

In this sense, although there are various software solutions, the Python programming language remains a solid basis to build such deep-learning systems based on artificial neural networks in the detection, identification or even monitoring of insect pest. Cloud computing services capable of providing modules, APIs or even software platforms as a service in the development of deep-learning solutions for pest detection have also been noted. The main characteristic in their case is represented by the availability and flexibility in accessing these types of cloud resources, being therefore part of the new trends.

Another software used for insect monitoring in precision and modern agriculture was Fastai. It is a high-level open-source DL library built on top of PyTorch (Howard & Gugger, 2020). It is designed to make it easier to train state-of-the-art DL models with as little code as possible. The library provides a simple and consistent API for quickly training deep NNs on a wide range of tasks, such as image classification, object detection, text classification, and natural language processing. One of the unique features of Fastai is its approach to transfer learning, which involves leveraging pre-trained models and fine-tuning them for specific tasks (Cochero et al., 2022).

Another modern software that was used for insect monitoring in agriculture was Imagga Cloud API which is a cloud-based image recognition platform that provides a suite of APIs for developers to build image-related applications (Imagga, 2020). Imagga API was used for rice pest detection (Bhoi et al., 2021), integrating IoT and UAV systems. The Imagga Cloud API provides a range of image analysis and recognition services, including image tagging, content-based image search, color extraction, cropping, and ML algorithms that can identify objects, scenes, colors, and other attributes within an image.

## 3 New trends in harmful insect and pest detection

Regarding insect monitoring for detection, classification, and even segmentation there are several modern approaches to train and validate a computer system for pest monitoring tasks using AI (Zhang, 2022). CNNs are frequently utilized in this procedure because they are particularly well-suited to image recognition tasks (Teng et al., 2022). Over time a well-trained system can be used to identify pests quickly and accurately in real-world scenarios and images, enabling farmers, growers, and other stakeholders to take action to address any issues quickly and effectively (Aota et al., 2021). The modification of networks in relation to specific detection or identification tasks has evolved over time and new ways of implementation and development have emerged to meet these needs.

Training and validating individual networks are the first starting point. Modifying existing architectures through various mathematical or structural methods is a common practice to increase the robustness of such a system. By increasing the number of training images and fine-tuning the network’s parameters, the accuracy of pest identification using NNs may be enhanced (Xia et al., 2018). This procedure is done multiple times until the system achieves a satisfactory level of accuracy (Butera et al., 2021). On the other hand, approaches to modify the base structure and new optimization methods are addressed to satisfy the same final need, to increase the accuracy and precision of a system in relation to representative areas for pest detection and monitoring. Models with notable results starting from the basic structures of state-of-the-art networks by applying transfer learning techniques, increasing dimensions, and implementing custom optimizations were developed (Abbas et al., 2021). The research papers adopted transfer learning applied to several public databases or similar research datasets noted and described in previous chapters. Oftentimes, research has involved the creation of proprietary and private databases that are focused on the needs of each area under investigation.

**Figure 6:**
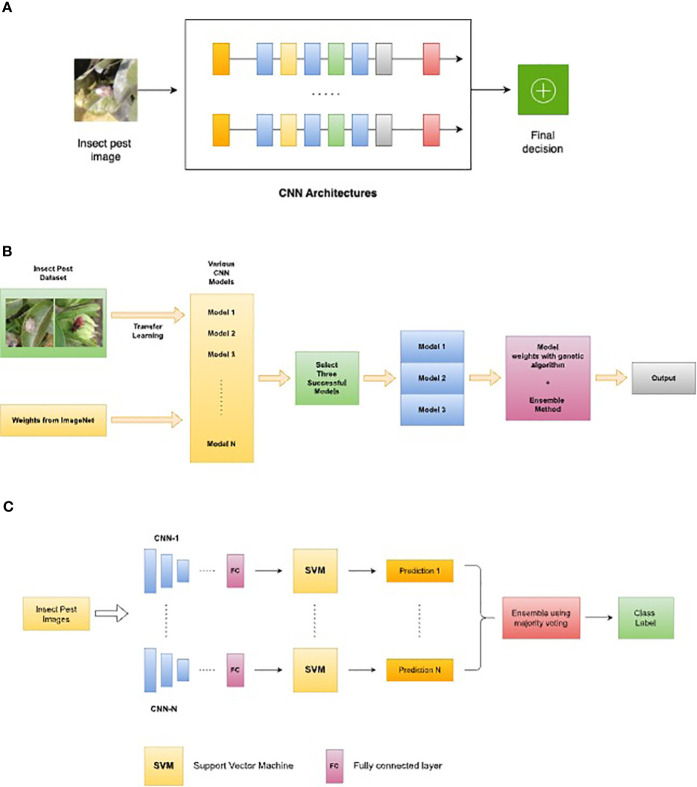
Examples of multi-network-based systems as a new trend: **(A)** Adapted system architecture for the ensemble model proposed in (Nanni et al., 2022) for insect pest detection, **(B)** Adapted insect classification ensemble methodology proposed in (Ayan et al., 2020), **(C)** Adapted majority voting ensemble model for pest classification proposed in (Turkoglu et al., 2022).

Multinetwork-based systems are new trends for insect monitoring and detection. The most representative ones are based on custom ensemble models. The use of ensembles of NNs and innovative modified architectures can improve the accuracy of pest detection. A CNN ensemble is a mixture of several CNN models that results in a stronger, more accurate prediction model. The aim of an ensemble is to use the strengths of many models to compensate for the shortcomings of a single model (Xu et al., 2021). The final decision of an ensemble of CNNs is derived by fusion of the predictions of separate CNN models, often by majority voting or weighted averaging. An ensemble’s diversity of models decreases the problem of overfitting, resulting in greater accuracy and precision. Once trained, the outputs of the individual CNN models are combined to form a final prediction. The idea is that by combining the predictions of multiple models, the overall accuracy and reliability of the system can be improved, and the risk of false positives or false negatives can be reduced. One of the main advantages of using a CNN ensemble for insect pest detection is that it can improve the ability of the system to generalize to new images or environments that may be different from the training dataset. By using multiple models with different strengths and weaknesses, the ensemble can be more robust to variations in lighting, background, or other factors that may affect the appearance of the insects in the images. The majority voting ensembles, weighted average ensembles, and multinetwork ensembles using a variety of CNNs backbones are the most popular and most adopted in the case of pest detection and identification. Some examples of ensemble models of NNs are presented in **Figure 6**.

Fusion by weighted sum rule and combinations based on different topologies and various Adam optimization were used (Nanni et al., 2022) for the detection of several insect pests attached to each database. The performance of the presented work was noted and compared for different datasets. CNN architectures are trained using various optimization functions, including some novel Adam variations, and then fused. The system is described in **Figure 6A** (inspired by Nanni et al., 2022). The paper compared some of the state-of-the-art architectures for pest classification: ResNet50, GoogleNet, DenseNet201, and EfficientNetB0. Some other models were added and used for their speed and efficiency on mobile devices: ShuffleNet and MobileNetV2. In terms of optimization, Adam variants like diffGrad was used to calculate a scaling factor in the learning rate.

Another strategy using transfer learning, fine-tuning, and model ensemble was proposed in (Ayan et al., 2020). D0, SMALL, and IP102 datasets were again selected and used to train, validate, and test the accuracy rates of the proposed models. The study involved modifying and re-training seven pre-trained CNN models using transfer learning and fine-tuning on a 40-class dataset.

The top three models (Inception-V3, Xception, and MobileNet) were ensembled using the sum of maximum probabilities and weighted voting with weights determined by a genetic algorithm to create two ensembled models: SMPEnsemble and GAEnsemble (Ayan et al., 2020). Pre-trained models on ImageNet were implemented and the proposed model of insect classification ensemble methodology can be seen in **Figure 6B**. The paper highlights that deep networks with different architectures can have varying generalization capabilities when trained on the same dataset. This is because different models can extract different features from the data based on their architecture. Therefore, it is important to consider the model architecture when selecting the best-performing model for a given task. Adopting the suitable CNN architecture for insect pest detection helps increase the detection system’s accuracy and efficiency. It may be able to construct models that are more adapted to certain pest detection tasks by using the inherent capabilities of each architecture because various insect pests might have diverse physical characteristics that necessitate specific detection procedures. Certain pests, for example, may have a distinguishing pattern of spots or stripes on their body, while others may have distinct antennae or wings. As a result, it is critical to carefully pick the CNN architecture to be employed for insect pest identification. Because of its capacity to extract data at different scales, an architecture like Inception-V3 may be more suited for pests with complex traits. MobileNet, on the other hand, can be more suited for simpler pests or resource-constrained applications because of its lightweight design.

Another ensemble model was proposed in (Turkoglu et al., 2022) using a majority voting method **Figure 6C**. Feature concatenation and SVM (Support Vector Machine) classifier was also implemented at the core of the proposed system which used six state-of-the-art networks for pest classification and plant disease classification.

The tendency of researchers to modify the NN backbone was also observed. Modifying the backbone of a pre-trained NN for a given task is a typical approach in deep learning (Li Z. et al., 2022). Many cutting-edge models are constructed on modified backbones of pre-trained NNs (Kuzuhara et al., 2020; Liu et al., 2022). Although this aspect does not define a new area, there are some directions that can be highlighted in relation to the idea of modifying the backbone of a neural network. In this sense, specific modifications of the backbone and the impact on the performance of the network can describe novel and innovative research (**Table 6**). Modifying the NN backbone can have a significant impact on its performance. For instance, changing the number of layers in the backbone can affect the depth of the network and its ability to learn more complex features. Adding or removing layers can also affect the number of parameters in the network, which can impact its overall computational efficiency. Additionally, changing the architecture of the backbone can impact the type of features extracted from the input data. A defining example of the area of innovation brought by modifying the backbone of a model can be researched in the study (Butera et al., 2021). The authors (Butera et al., 2021) used Faster R-CNN, SSD, and RetinaNet. Backbone used was based on several models such as VGG, ResNet, DenseNet, and MobileNet, adapted for the task of insect pest detection in real-world scenarios. Additionally, the impact of the transfer learning technique on the models used for accuracy and inference time was also studied. The authors noted that a model based on Faster R-CNN with MobileNetv3 is a strong point for insect pest detection.

**Table 6:** CNN ensemble architectures and backbone modifications.

NN Used	Novelty	Combination/Description	Function/ Application	Perfor-mances	Papers, year
AlexNet, GoogleNet, DenseNet201	CNN Ensemble	▪ Majority voting fusion	Classification/Apple pest and disease classification in a real-time application	ACC: 96.1% -99.2%	(Turkoglu et al., 2020)
AlexNet, VGG16, ResNet-50, InceptionResNet V2	CNN Ensemble	▪Fusion by correlation coefficient comparison ▪Majority voting	Classification/Citrus pest	F1 Score: 0.935	(Khanramaki et al., 2021)
EfficientNetB0, GoogleNet, ResNet-50, MobileNetV2, ShuffleNet, DenseNet201	CNN Ensemble	▪Fusion by weighted sum rule ▪Combination based on different topologies ▪ Various Adam optimization	Detection/ Insect pest	ACC: 95.52% (SMALL), 74.11% (IP102), 99.81% (D0)	(Nanni et al., 2022)
AlexNet, ResNet 18, 50 and 101, DenseNet201, GoogleNet	CNN Ensemble	▪ Fusion by averaging ▪ Majority voting ▪Integrating SVM classifier	Detection and classification/Plant disease and pest	ACC: 97,56%, 96.83%	(Turkoglu et al., 2022)
Inception-V3, ResNet-50, Xception, VGG-16, VGG-19, MobileNet	CNN Ensemble	▪Fusion by majority voting ▪Four-stage classification methodology	Classification/Insect	ACC: 98%	(Ayan et al., 2020)
FasterRCNN, MobileNetV3	Backbone modification	▪FasterRCNN with MobileNetV3 backbone	Detection/Insect pest	mAP: 92.66%	(Butera et al., 2021)
YOLO-v4-tiny, CSPDarknet53	Backbone modification	▪YOLO v4-tiny with CSPDarknet53-tiny backbone	Detection/Insect pest	F1: 0.838	(Genaev et al., 2022)
R-CNN ResNet50	Backbone modification	▪Novel MSR-RCNN model with ResNet-50 backbone	Detection/ Multi-class pest	mAP: 67.4%	(Teng et al., 2022)
SSD, RetinaNet, FCOS, R-CNN, FPN, Cascade R-CNN	Backbone modification	▪SSD with VGG16 as backbone ▪ResNet 50 for object detetion	Detection/Insect pest		(Wang et al., 2022)
RetinaNet	Backbone modification	▪RetinaNet with feature pyramid network backbone	Detection/multi-scale insect detector	mAP: 94.77%	(Li et al., 2020)
VGG, ZFNet, ResNet 50 - 101, Faster R-CNN	Backbone modification	▪Deep CNN fused with CSA	Detection and classification/ Multi-class pests	mAP: 75.46%	(Liu et al., 2019)
YOLOv3, Xception	Two-stage detector	▪Two-stage detection using YOLOv3 and Xception	Detection and classification/ Small insect pests	PRE: 77%	(Kuzuhara et al., 2020)
Inception, ResNet50	Two-stage detector and backbone modification	Two-stage CNN solution integrating GaFPN and GAM	Detection and classification/ Small insect pests	mAP: 71%	(Liu et al., 2022)
YOLOv5, ShuffleNetv2	Model combination	ShuffleNetv2-YOLOv5-Lite-E improved detection model for edge devices	Detection/ Tea culture pest	mAP: 97,43%	(Zhang et al., 2023)
GhostNet, YOLOv5	Model combination	YOLOv5-GhostNet combination for embedding devices	Detection/ Orchard pest	mAP: 99%	(Zhang Y. et al., 2022)

The YOLOv4-tiny architecture with CSPDarknet53-tiny as the backbone was used to train a pest fly detection model using a dataset of insects of interest (Genaev et al., 2022). The network consists of Backbone, Neck, and three recurring blocks including Convolution, CBL, and CSP blocks. The CSP block structure utilizes a feature pyramid network to divide the input feature map into two parts. This structure reduces computational complexity while maintaining accuracy in object detection. Using YOLOv4-tiny allowed for the development of a fly recognition method that can be implemented as a modern mobile application.

A network for robust pest detection, with emphasis on small-size, multi-scale, and high-similarity pests was proposed by the authors (Teng et al., 2022). The proposed pest detection network used two customized core designs: a multi-scale super-resolution (MSR) feature enhancement module and a Soft-IoU (SI) mechanism. The MSR module developed enhances feature expression ability for small-size, multi-scale, and high-similarity pests, while the SI mechanism emphasizes position-based detection requirements. The MSR-RCNN is more suitable for pest detection tasks and includes a ResNet50 backbone and a feature full fusion mechanism to improve multi-scale pest detection. A feature full weighting mechanism was added and optimizes the detection performance of similar pests from two dimensions (depth and location). The implemented MSR module includes a super-resolution component used to obtain a six-layer feature map for recognizing small-sized objects. Additionally, the full feature fusion mechanism is used to integrate all features at once for recognizing multi-scale objects. On the other hand, in this study, a large-scale pest dataset of trap images was developed (LLDP-26). It can be observed that the changes made to the existing models and backbones bring considerable improvements in performance, enabling the solution of pest identification problems from digital images and outperforming existing state-of-the-art models and techniques.

A two-stage detection and identification method for small insect pests using CNN was proposed in (Kuzuhara et al., 2020). The authors used YOLOv3 as an object detection model, which is a popular deep learning model for object detection. A region proposal network (RPN) to help identify the regions of the image that contain the pest is used. After identifying the regions of interest, the proposed method performs pest classification using the Xception model (Chollet, 2017), which is a deep CNN that has been shown to achieve high accuracy in image classification tasks. The authors further improved the classification accuracy by using a data augmentation method based on image processing, which helped to generate more training examples by applying transformations to the original images. One of the strengths of this two-stage detection method is that it can handle the challenges posed by small insect pests, which are difficult to detect using traditional object detection methods due to their small size and low contrast. This method shows a good way in achieving high accuracy in detecting and identifying small insect pests, which can help improve pest management in agriculture.

Regarding the new trends, the authors in (Zhang et al., 2023) proposed an improved detection model based on ShuffleNetv2 and YOLOv5. This paper presents a target detection model based on the ShuffleNetv2-YOLOv5-Lite-E method, which substitutes the Focus layer with the ShuffleNetv2 algorithm. It also reduces the model size by pruning the YOLOv5 head at the neck layer. The suggested model is more robust and lightweight, and it may enhance detection efficiency while maintaining the recognition rate.

Combining YOLOv5 and GhostNet (Zhang Y. et al., 2022) and using a custom pest dataset allowed the method to achieve a higher mAP with the same number of epochs. In this case, the usage of GhostNet in YOLOv5 can be described as a new trend. GhostNet is a lightweight neural network architecture proposed in 2020 (Han et al., 2020) for usage in edge devices. The utilization of Ghost modules, which replace the usual convolutional layers in a NN, is the core characteristic of GhostNet. Ghost modules have a primary and secondary path. The primary path is a normal convolutional layer, but the secondary path has fewer channels and is used to simulate the behavior of the primary path. GhostNet may achieve equivalent precision to bigger networks by employing Ghost modules but with fewer parameters and lower processing cost. This makes it appropriate for deployment on low-power devices with limited processing resources. For the proposed model, authors noted 1.5% higher mAP than the original YOLOv5, with up to three times fewer parameters and the same or less detection time. With this architecture, the mAP obtained by the authors was about 99%.

**Table 6** synthesizes the novelty and performances of CNN ensemble architectures.

## 4 Applications

In real applications, data classification and analyzing huge volumes of data are time-consuming. To increase efficiency, the final strategy is to create and optimize ML and DL models to estimate and create powerful systems for understanding features, patterns, and complex, big amounts of data (Csillik et al., 2018; Abayomi-Alli et al., 2021). The focus area is to train models to find optimal parameters, auto-adjust values, and adapt to a robust architecture generated and optimized step by step over several epochs of training with dataset capture (Nanni et al., 2022; Wang et al., 2022). For agricultural areas, ML is widely used to automate time-consuming, labor-intensive tasks and to collect essential information having at the core mathematical models, computational resources, and infrastructure with high performance and standards. As part of this study, we can note this as a new trend in precision agriculture. Proposed works show considerable results and note the popularity of AI in general. The applicability aspect of using these defined systems brings to the forefront a series of advantages and development areas. As can be seen from **Table 7**, most of the papers are focused on the following main applications: harmful insect detection, identification of infected crops, and crop monitoring.

**Table 7:** Applications.

Application	Papers
Harmful insect detection	(Albanese et al., 2021), (Alsanea et al., 2022), (Ayan et al., 2020), (Butera et al., 2021), (Cochero et al., 2022), (Genaev et al., 2022), (Guo et al., 2021), (Hansen et al., 2019), (Hong et al., 2021), (Hossain et al., 2019), (Iost Filho et al., 2022), (Espinoza et al., 2016), (Kasinathan et al., 2021), (Khanramaki et al., 2021), (Knyshov et al., 2021), (Li et al., 2019), (Li et al., 2020), (Li C. et al., 2022), (Liu et al., 2019), (Liu and Wang, 2020), (Lv et al., 2022), (Malathi and Gopinath, 2021), (Nagar and Sharma, 2021), (Nanni et al., 2022), (Rajeena et al., 2022), (Rimal et al., 2022), (Rustia et al., 2020), (Sanghavi et al., 2022), (Teng et al., 2022), (Valan et al., 2019), (Wang et al., 2020), (Wang et al., 2022), (Xia et al., 2018), (Zhang & Chen, 2020), (Shi et al., 2020)
Infected crops by insects	(Bereciartua-Pérez et al., 2022), (Bhoi et al., 2021), (Fang et al., 2020), (Espinoza et al., 2016), (Kusrini et al., 2021), (Nazri et al., 2018), (Sharma et al., 2020), (Singh et al., 2021), (Tian G. et al., 2020), (Turkoglu et al., 2020), (Turkoglu et al., 2022), (Wu et al., 2019), (Xing et al., 2019), (Xu et al., 2022), (Zhang S. et al., 2022), (Zhu et al., 2020)
Crop monitoring	(Ahmad et al., 2021), (Aota et al., 2021), (Bouroubi et al., 2018), (Brunelli et al., 2020), (De Cesaro Júnior et al., 2022), (Ding & Taylor, 2016), (Dai et al., 2021), (Partel et al., 2019), (Dos Santos et al., 2022), (Takimoto et al., 2021), (Zhong et al., 2018)

### 4.1 Harmful insect detection

CNNs have become increasingly popular in image-processing applications for modern agriculture following their ability to identify insects and features in images. According to this study, one of the applications of CNNs in the field of modern and precision agriculture is harmful insect detection. The identification of harmful insects is crucial for the protection of crops and the prevention of plant diseases (Lv et al., 2022). CNNs can be an effective tool for harmful insect detection in images (Guo et al., 2021; Alsanea et al., 2022). By training the network on a large and diverse dataset, CNN can learn to identify a wide range of harmful insects. However, the issues of class imbalance and transferability need to be addressed to ensure that CNN performs well in real-world applications. For effective detection of harmful insects, the first step is to collect and label a dataset of digital images containing both harmful and non-harmful insects (Cochero et al., 2022; Wang et al., 2022). In this case, the dataset should be large and diverse to ensure the great performance of the CNN model and to ensure that the CNN can learn to recognize a wide range of harmful insects. Because this detection uses CNN models that learn different features of an image through convolutional operations, the second step is the preprocess the images in the dataset created to ensure that they are in a format that can be fed into the CNN. This may involve resizing the images, converting them to grayscale, or normalizing the pixel values (Alsanea et al., 2022; Zhang Y. et al., 2022). Following this scenario, the next step is to train the chosen CNN model using the dataset prepared (Malathi and Gopinath, 2021; Nagar and Sharma, 2021; Liu et al., 2022). This involves feeding the network the labeled images and adjusting the weights of the neurons through backpropagation to minimize the error between the predicted and actual labels. Transfer learning applied on a custom insect pest dataset can be used and hyperparameter tuning to speed up the process in this topic. Related to this aspect, most of the papers analyzed for this study include such methodology (Ayan et al., 2020). After the CNN was trained, it can be used to classify new images of insects as either harmful or non-harmful. To do this, the new image is fed into the CNN, and the output is a probability score indicating the likelihood that the insect in the image is harmful. A threshold value can be set, and if the probability score is above this value, the insect is classified as harmful.

One of the main challenges that were identified in applications for harmful insect detection using CNNs is the issue of class imbalance (Du et al., 2022). Harmful insects may be rare in the dataset, which can lead to the CNN being biased towards non-harmful insects. To overcome this, techniques such as over-sampling or under-sampling can be used to balance the dataset. Another challenge identified is the issue of transferability. CNNs trained on one dataset may not perform well on a different dataset due to differences in the types of insects or the background images. To address this, transfer learning can be used, which involves using a pre-trained CNN as a starting point and fine-tuning the network on the new dataset, as mentioned earlier (Butera et al., 2021; Li W. et al., 2022; Popkov et al., 2022).

### 4.2 Infected crops by insects

CNNs are a powerful tool for identifying insect-infected crops. They can be trained to learn patterns and features in images that are indicative of insect damage and provide predictions on whether the crops are healthy or infected (Turkoglu et al., 2022; Zhang S. et al., 2022). The use of CNNs in agriculture can improve crop yields and help farmers prevent and manage insect infestations more effectively (Espinoza et al., 2016; Sharma et al., 2020; Bereciartua-Pérez et al., 2022). Infected crops by insects can have a significant impact on the agricultural industry, leading to the loss of crops and revenue (Xu et al., 2022). With the increasing advancements in computer vision, for modern agriculture, our study highlights that the CNNs became an effective tool for identifying and detecting insect infestations in crops.

CNNs are commonly utilized in applications such as image classification, object identification, and segmentation. CNNs may be taught to recognize patterns and characteristics in images that are indicative of insect damage in the context of recognizing insect infestations in crops. Similarly, to the insect detection tasks discussed, a huge collection of images of healthy and infected crops must be developed for applications used to target diseased crops. The images are then annotated with whether the crops are healthy or sick, as well as the species of bug inflicting the harm. The CNN models are then trained by giving them tagged images, allowing them to understand the patterns and characteristics associated with insect-infested crops.

On the other hand, the CNN model can also provide information about the type of insect causing the damage, enabling farmers to take appropriate measures to prevent further damage. In addition to identifying insect-infected crops, CNNs can also be used for segmentation tasks (Zhang & Chen, 2020). Segmentation involves dividing an image into different regions or objects. In the context of identifying insect infestations, segmentation can be used to identify and evaluate the areas of the crop that are infected. This can provide more detailed information to farmers and enable them to target their treatment strategies more effectively.

### 4.3 Crop monitoring

The third area of applications using models based on DL, respectively on CNNs, is crop monitoring. This area of application has a major impact when considering pest insect populations and managing the effects of their presence. In this sense, there have been several studies that have included this direction of development. Crop monitoring refers to the process of keeping track of the growth, development, and health of crops. Crop monitoring can be done using a variety of methods, including satellite imagery, drone imagery, ground-based sensors, and visual inspections. However, the traditional methods of crop monitoring can be time-consuming, expensive, and require a significant number of resources. With the advent of AI and ML, the use of CNNs for crop monitoring has become increasingly popular. In crop monitoring, CNNs can be used to analyze images of crops and provide insights into their growth, development, and health. The process of crop monitoring using CNNs typically involves several steps including data collection, data preprocessing, training NNs, and evaluating performances in a specific area of interest (Dai et al., 2021). Applications of crop monitoring using CNNs have a wide range of applications in modern agriculture, including disease and pest detection or even yield estimation (Zhong et al., 2018; Tian G. et al., 2020). CNNs can be used to detect the presence of diseases in crops by analyzing the images of the leaves and other parts of the plant. This can help farmers to take timely action to prevent the spread of diseases and minimize crop losses. On the other hand, this process can be automated by introducing real-time monitoring modules, based on hardware systems and software modules optimized for mobile platforms, used in the field. Important to note, crop monitoring using CNNs has the potential to revolutionize agriculture by providing farmers with real-time insights into the growth, development, and health of their crops. CNNs can analyze images of crops quickly, accurately, and at a fraction of the cost of traditional methods (Vanegas et al., 2018). By using CNNs for crop monitoring, farmers can make informed decisions about crop management, minimize losses due to meteorological conditions, diseases, and pests, and optimize their yields.

## 5 Discussion

This review paper points out several features in relation to the areas of massive pest detection, classification, and recognition in various crops. The research method plans to highlight the advantages and disadvantages as well as the new trends of CNNs and the application of image processing within these aspects of PA. On the other hand, this study highlights the use of innovative approaches and techniques, such as DL, transfer learning, active learning, ensembles of CNNs, and multi-scale feature fusion, for pest detection and classification from digital images. Overall, this study is focused on insect monitoring including real environment, NNs, and new trends.

Harmful insects and pest detection present a series of challenges that researchers tend to study more and more and solve the problems that arise. Analyzing the research extracted from established databases, we noticed the wide interest in recent years based on the topic of modern and precision agriculture. As it was presented in the previous chapters, the databases chosen for extracting the papers of this review study were Web of Science, IEEE, and Scopus. Most of the papers chosen for analysis were extracted from the Web of Science database one of the most widely used citation databases in the world. Research on new trends and impact information has been placed in the 2020-2022 range. For the review topic, similar articles were extracted and compared. Their analysis is presented in **Table 8**, where the differences compared to this presented review and the area of contributions were also noted. Based on the analysis, good quality information was highlighted, and it was observed that the interest in the detection of harmful insects and pests in modern agriculture using image processing and NNs is quite pronounced.

**Table 8:** Recent review/survey papers on similar topics.

Paper/ year	Description	Period	Refe-rences	Our differences
(Abade et al., 2021)	▪ Systematic plant disease review ▪ CNN for crop disease recognition – trends and gaps. ▪ State of the art through systematic review used – StArt Tool	2010-2019	121	▪ Focused on insects including real environment. ▪ Focused on new trends (including 2022). ▪ New investigated methods for review papers (PRISMA). ▪ More references.
(De Cesaro Júnior & Rieder, 2020)	▪ Different approaches like CNN and other image classifiers for insect or diseased plants detection from images.	2015 - 2019	57	▪ Focused on insects including real environment. ▪ Focused on new trends (including 2022). ▪ New investigated methods for review papers (PRISMA). ▪ More references.
(Cardim Ferreira Lima et al., 2020)	▪ Identification and monitoring of insect pests using automatic traps. ▪ Using infrared sensors, audio sensors, and image-based classification	2007 - 2020	77	▪ Focused on more insects including real environment ▪ Focused on image processing ▪ Focused on neural networks ▪ Focused on new trends (including 2022). ▪ More references. ▪ New investigated methods for review papers (PRISMA)
(Iost Filho et al., 2019)	▪ Using drones in pest management to obtain canopy reflectance data of arthropod infested plants.	1998 - 2018	319	▪ Focused on insects including real environment ▪ Focused on new trends (including 2022). ▪ Focused on neural networks ▪ New investigated methods for review papers (PRISMA)
(Ghosh et al., 2021)	▪ Strategies and future trends on molecular and automated pest identification (thrips) for rapid and high throughput diagnosis.	2001-2020	253	▪ Focused on insects including real environment ▪ Focused on new trends (including 2022). ▪ New investigated methods for review papers (PRISMA)
(Kumar & Kukreja, 2022	▪ Systematic review on wheat disease prediction models Kitchenham investigation method (Kitchenham et al., 2010)	1997-2021	102	▪ Focused on insects including real environment ▪ Focused on new trends (including 2022). More references. New investigated methods for review papers (PRISMA)
(Liu & Wang, 2021)	▪ Plant disease and pest detection based on deep learning ▪ Aspects of classification, detection and segmentation networks are discussed	2014-2020	108	▪ Focused on new trends (including 2022). ▪ Focused on insects including real environment ▪ More references. ▪ New investigated methods for review papers (PRISMA)
(Preti et al., 2021)	▪ Insect pest management using camera-equipped traps and smart traps ▪ Remote sensing and electronics for long-distance pest monitoring ▪ Automatic detection and analysis for insect detection and counting ▪ Automatic traps usage benefits	1980-2020	75	▪ Focused on new trends (including 2022). ▪ Focused on image processing ▪ Focused on neural networks ▪ More references. ▪ New investigated methods for review papers (PRISMA)
(Toscano-Miranda et al., 2022)	▪ Insect pests and disease detection in cotton cultures using ML and IoT ▪ Focused on remote sensing and AI techniques ▪ Trends for smart agriculture ▪ Kitchenham investigation method [Kit 10]	2014-2021	100	▪ Focused on new trends (including 2022). ▪ Focused on insects including real environment ▪ Focused on image processing ▪ Focused on neural networks ▪ More references ▪ New investigated methods for review papers (PRISMA)

Training, validation, and testing modalities are important points in the research of architectures that automate processes in modern agriculture. In the initial steps, acquiring the data set and organizing it is extremely important. Most papers reviewed for this study highlighted the impact of a robust dataset, adding images taken from real contexts. It has been observed that for the modern area, techniques such as data augmentation and synthetic data generation play an important role to diversify the data set. These implications solve the problems where the training and validation data set is small and for multi-class pest detection tasks it can solve the class imbalance problem. A modern use case was noted by the authors in (Karam et al., 2022) developing a web app for synthetic data generation using DC-GANs, for agricultural pest detection (whiteflies). The study illustrates how employing GAN in the pipeline can improve the model’s capacity to generalize and hence improve the accuracy of detected bounding boxes.

Image processing is another important step to note. Due to the acquisition of digital images from real contexts, the presence of insects at the image level presents some aspects that have a negative impact on the training and evaluation of the model that receives this data as input. These aspects are represented by the relatively small size of the insects, artifacts at the image level, and the context in which they are illustrated: complex background, various types of occlusions (branches, leaves), the presence of insects in large numbers, and small object detection. Image processing aids in the preprocessing and enhancement of input pictures, hence boosting the accuracy and performance of CNN models. Images collected from various sources, such as digital cameras or drones, may have differences in lighting, background noise, and other artifacts that might affect the accuracy of insect detection. As a result, image processing techniques like filtering, segmentation, and normalization can aid in the removal of noise and artifacts, the improvement of contrast, and the highlighting of areas of interest in pictures. Image processing may also aid in the extraction and selection of useful aspects from digital images, such as color, texture, and shape, that are significant to insect pest identification. The CNN models can learn to discriminate between various insect species and effectively categorize them by finding and extracting these traits, even in complex situations.

To synthesize the findings, the present review paper highlighted the fact that the combination of CNN architectures, as well as the modification of existing architectures through various techniques, bring to the fore notable performances in terms of accuracy. According to the previously mentioned characteristics related to the novelty in the combination of convolutional neural networks and the problems in the detection of harmful insects of interest, a series of studies of interest were identified with various presented methods and integrating databases illustrating real contexts.

Starting in 2019, the authors (Liu et al., 2019) presented a DL approach named PestNet. It was highlighted that multi-class pest detection is a crucial step for effective pest management in modern agriculture. In this work, PestNet includes a novel channel-spatial attention module, a region proposal network, and a position-sensitive score map (PSSM). A newly collected large-scale pest image dataset named MPD2018 was proposed to evaluate the PestNet model achieving 75.46% mAP on 16 pest classes, outperforming other state-of-the-art methods.

Following Pest24 paper and database, to evaluate multi-pest detection performance, the dataset described is divided into training, validation, and test sets, with four state-of-the-art object detection methods employed. YOLOv3 achieves the highest mAP of 63.54% and an impressive AP of 98.6% for individual pests under optimal parameters. A 3-fold cross-validation experiment confirms similar results. The paper examines various factors affecting detection performance, highlighting the significant impact of relative scale on AP while indicating that color discrepancy has negligible influence.

Authors (Wang et al., 2022) also proposed a DL model, this time for the recognition and counting of apple pests. The MPest-RCNN named model achieved mAP and F1-Score values of 99.11% and 99.50%, evaluated using an original dataset of three typical pests in apple orchards. The paper presents a new Faster R-CNN structure based on the ResNet101 feature extractor and a novel CNN structure with small anchors to extract features, therefore boosting recognition accuracy for small pests.

Hunger Games search-based deep convolutional neural network (HGS-DCNN) model for crop pest image classification was proposed (Sanghavi et al., 2022), adding a new convolutional layer to decrease parameter redundancy. Pre-processing and augmentation, followed by pest categorization, are the two steps of the model proposed. Pre-processing makes use of a novel adaptive cascaded filter (ACF) in conjunction with decision-based median filtering (DMF) and guided image filtering techniques. The proposed model outperformed existing pre-trained architectures such as ResNet50, EfficientNet, Dense Net, Inceptionv3, and VGG-16 in terms of accuracy, precision, F1-score, sensitivity, and specificity, with values of 99.1%, 97.80%, 97.80%, 97.82%, and 99.43%, respectively.

In the area of precision agriculture, the advent of new-generation AI technology has ushered in a promising era of real-time pest population monitoring. CNNs have exhibited amazing performance in insect pest identification and categorization as part of deep learning approaches. Their capacity to learn detailed characteristics from large-scale visual data permits accurate recognition, even when inter-class variances are small. Factors like as dataset size, model design, and data quality can all have an impact on CNN performance. It is still difficult to provide robustness against intra-class volatility and data imbalance. Ongoing research in pest identification and monitoring enhances CNNs’ capabilities. Collaboration among agricultural, entomology, computer vision, and machine learning professionals enables transdisciplinary solutions.

## 6 Conclusions

Following this study, the use of new trends in deep learning has the potential to revolutionize the field of pest monitoring and significantly improve pest management in agricultural sector. Algorithms such CNNss have shown great promise in accurately identifying and classifying pests in digital images with high precision and accuracy rates. Currently, CNNs have become a potent tool in identifying crops that are infected with insects. Researchers have developed ensemble techniques where multiple CNN models are combined to achieve better performance. This technique is becoming increasingly popular in the field of pest identification due to its effectiveness in handling complex datasets and the ability to capture diverse features of insects. Optimizing existing models for identifying harmful insects by modifying their architectures specifically for this topic represents another approach with a strong innovative impact. For modern and precision agriculture or integrated pest management, farmers can enhance their treatment approaches by utilizing applications like insect detection for harmful insects, identifying crop infections caused by insects, or monitoring crop growth, which can offer them comprehensive insights and allow them to precisely target their treatments.

## Author contributions

DP: Writing – original draft, Writing – review & editing, Conceptualization. AD: Writing – original draft, Writing – review & editing, Methodology. LI: Writing – original draft, Writing – review & editing, Formal Analysis, Investigation. NA: Formal Analysis, Writing – original draft, Writing – review & editing.
